# Another voice in the crowd: the challenge of changing family planning and child feeding practices through mHealth messaging in rural central India

**DOI:** 10.1136/bmjgh-2021-005868

**Published:** 2021-07-26

**Authors:** Kerry Scott, Osama Ummer, Aashaka Shinde, Manjula Sharma, Shalini Yadav, Anushree Jairath, Nikita Purty, Neha Shah, Diwakar Mohan, Sara Chamberlain, Amnesty Elizabeth LeFevre, Kilkari Impact Evaluation team, Smisha Agarwal

**Affiliations:** 1 Department of International Health, Johns Hopkins Bloomberg School of Public Health, Baltimore, Maryland, USA; 2 Oxford Policy Management, New Delhi, India; 3 BBC Media Action, New Delhi, India; 4 Independent researcher, New Delhi, India; 5 School of Public Health and Family Medicine, University of Cape Town, Cape Town, South Africa

**Keywords:** child health, health education and promotion, health services research, health systems, public health

## Abstract

**Introduction:**

Kilkari is one of the world’s largest mobile phone-based health messaging programmes. Developed by BBC Media Action, it provides weekly stage-based information to pregnant and postpartum women and their families, including on infant and young child feeding (IYCF) and family planning, to compliment the efforts of frontline health workers. The quantitative component of a randomised controlled trial (RCT) in the Indian state of Madhya Pradesh found that exposure to Kilkari increased modern contraceptive uptake but did not change IYCF practices. This qualitative research complements the RCT to explore why these findings may have emerged.

**Methods:**

We used system generated data to identify households within the RCT with very high to medium Kilkari listenership. Mothers (n=29), as well as husbands and extended family members (n=25 interviews/family group discussions) were interviewed about IYCF and family planning, including their reactions to Kilkari’s calls on these topics. Analysis was informed by the theory of reciprocal determinism, which positions behaviour change within the interacting domains of individual attributes, social and environmental determinants, and existing practices.

**Results:**

While women who owned and controlled their own phones were the Kilkari listeners, among women who did not own their own phones, it was often their husbands who listened. Spouses did not discuss Kilkari messages. Respondents retained and appreciated Kilkari messages that aligned with their pre-existing worldviews, social norms, and existing practices. However, they overlooked or de-emphasised content that did not. In this way, they reported agreeing with and trusting Kilkari while persisting with practices that went against Kilkari’s recommendations, particularly non-exclusive breastfeeding and inappropriate complementary feeding.

**Conclusion:**

To deepen impact, digital direct to beneficiary services need to be complimented by wider communication efforts (e.g., sustained face-to-face, media, community engagement) to change social norms, taking into account the role of socio-environmental, behavioural, and individual determinants.

**Key questions**

**What is already known?**

- A randomised controlled trial in Madhya Pradesh found that exposure to Kilkari mobile health information messages was associated with an increase in modern reversible contraceptive use.
- However Kilkari was not found to have an impact on infant and young child feeding (IYCF) practices, including exclusive breastfeeding and complementary feeding practices.
- This qualitative research examines the factors underpinning the quantitative findings by assessing who, within the household, picked up and listened to Kilkari calls and how families interpreted Kilkari messages on family planning and IYCF.

**Key questions**

**What are the new findings?**

- Reaching women who do not own their own phones is a challenge because incoming calls made to ‘shared’ phones are often picked up by husbands; moreover, spouses reported very rarely or never discussing the contents of Kilkari messages.
- Respondents retained and appreciated Kilkari call content that aligned with existing practice, social norms, and worldview, for example, to breastfeed, give homecooked food once complementary feeding is initiated, have a small family, and immunise the child.
- Listeners generally failed to absorb or overlooked messages that went against practice, social norms, and worldviews, for example, not to give water or health tonics to the infant during the first 6 months, and to give only thick foods once complementary feeding commences—even messages on the safety and effectiveness of modern contraception were rejected by many respondents.

**What do the new findings imply?**

- Health behaviour change encouraged by mHealth messaging must be bolstered by face-to-face counselling and efforts to create a more enabling social environment.

## Introduction

Despite steady improvement, suboptimal infant and young child feeding practices and low uptake of reliable family planning methods continue to drive morbidity and mortality and curtail life opportunities worldwide. Non-exclusive breastfeeding has been estimated to underpin up to 13% of global under-five mortality^1^ and undernutrition is associated with 45% of child deaths.^2^ As of 2017, global unmet need for family planning^1^ remained above 20%.^3^

In India’s economically deprived central and northern states, including the central state of Madhya Pradesh where this research took place, nutrition and family planning indicators showcase ongoing challenges. The prevalence of stunting, wasting, and underweight in Madhya Pradesh among children below 5 years remains high at 40%, 20%, and 39%, respectively, indicating the existence of both chronic and acute malnutrition.^4^ The 2015 National Family Health Survey 4 (NFHS-4) found that only 58% of the babies under 6 months of age were exclusively breastfed, only 38% of children 6–8 months in age were receiving both breast milk and complementary foods, and just 7% of children 6–23 months in age received adequate dietary diversity and meal frequency.^5^ The contraceptive prevalence rate among married women of reproductive age in Madhya Pradesh was 51% in 2015 (NFHS-4) and approximately 14% of women had an unmet need for contraception.^6^

While child nutrition and family planning are grounded in socio-economic and environmental determinants, educational interventions focused on improving women’s knowledge and attitudes have improved child feeding behaviour and nutritional outcomes^7–10^ and increased uptake of modern contraceptives.^11 12^ With the increasing global prevalence of mobile phones, mHealth programmes have the potential to bring health content directly to millions of people through audio or text messages on their phones. Despite their proliferation, there is only limited evidence that mHealth interventions can improve aspects of routine health behaviour^13^ and can specifically enhance child feeding^14 15^ and contraception use.^16^ There is little known about engagement with mHealth messages by the user (e.g., who listens, whether messages are discussed) and how users comprehend and reconcile the messages in relation to their own ongoing practices, perspectives, and social worlds.

### The Kilkari mHealth programme and evaluation

Kilkari is the Government of India’s flagship mHealth messaging programme. Designed and scaled by BBC Media Action in collaboration with the Ministry of Health and Family Welfare, Kilkari had reached 10 million subscribers in 13 states by December 2018.^17^ The weekly calls consist of approximately 1.5 min of pre-recorded audio content delivered by a fictitious female character called Dr Anita. Kilkari enrolment can occur as early as the fourth month of pregnancy and the programme ends at the child’s first birthday. The calls, which address both mothers and fathers, are stage-based, meaning that each call is tailored for the specific stage of pregnancy or postpartum when it is delivered. The messages cover 11 reproductive, maternal, neonatal, and child health (RMNCH) behaviours, and have the greatest portion of content on family planning (16% of the total content) and infant and young child feeding (IYCF) (13%) (see online supplemental file 1 for the messages).

10.1136/bmjgh-2021-005868.supp1 Supplementary data

Kilkari is to complement the face-to-face behaviour change communication efforts of frontline health workers (FLHWs), particularly accredited social health activist (ASHA) community health workers, auxiliary nurse midwives, and anganwadi nutrition workers.^18^ Most messages include encouragement that listeners reach out to their FLHWs for more information. Kilkari was developed in Bihar, using principles of human-centred design,^19^ as part of a suite of complementary mobile health services, which included Mobile Kunji. Mobile Kunji was a job aid for FLHWs that used audio messages from Dr Anita and a printed deck of cards to facilitate face-to-face communication between FLHWs and families on the same topics covered in Kilkari. However, despite evidence of its positive impact on key RMNCH behaviours,^20^ Mobile Kunji was not scaled up.

The Kilkari Impact Evaluation was a randomised controlled trial (RCT) that enrolled 5095 pregnant women in rural Madhya Pradesh.^21^ It found that exposure to Kilkari was associated with higher reported usage of modern reversible contraception: instrumental variable analysis found that current use of modern reversible contraceptives (primarily condoms and oral contraceptive pills) was 31% among unexposed and 38% among those exposed.^21^ Kilkari also had a positive impact on one other practice (immunisation at 10 weeks) and nine knowledge and decision-making indicators but did not have an impact on any other health practices, including IYCF practices.^21^

The qualitative research presented here examines how Kilkari calls enter a social world of relationships and pre-existing norms that facilitate or hinder message reach and impact. Data automatically generated by the Kilkari system tells us whether each incoming call was picked up, and how long the message played before the call was disconnected; this qualitative research explores who actually picks up and listens to the calls and why calls were not picked up or cut. This qualitative research also suggests why the impact evaluation outcomes occurred by illuminating listener perspectives on Kilkari, interpersonal communication in relation to Kilkari, and barriers and facilitators to the adoption of Kilkari’s recommended practices.

### Conceptual framework

**Figure 1:**
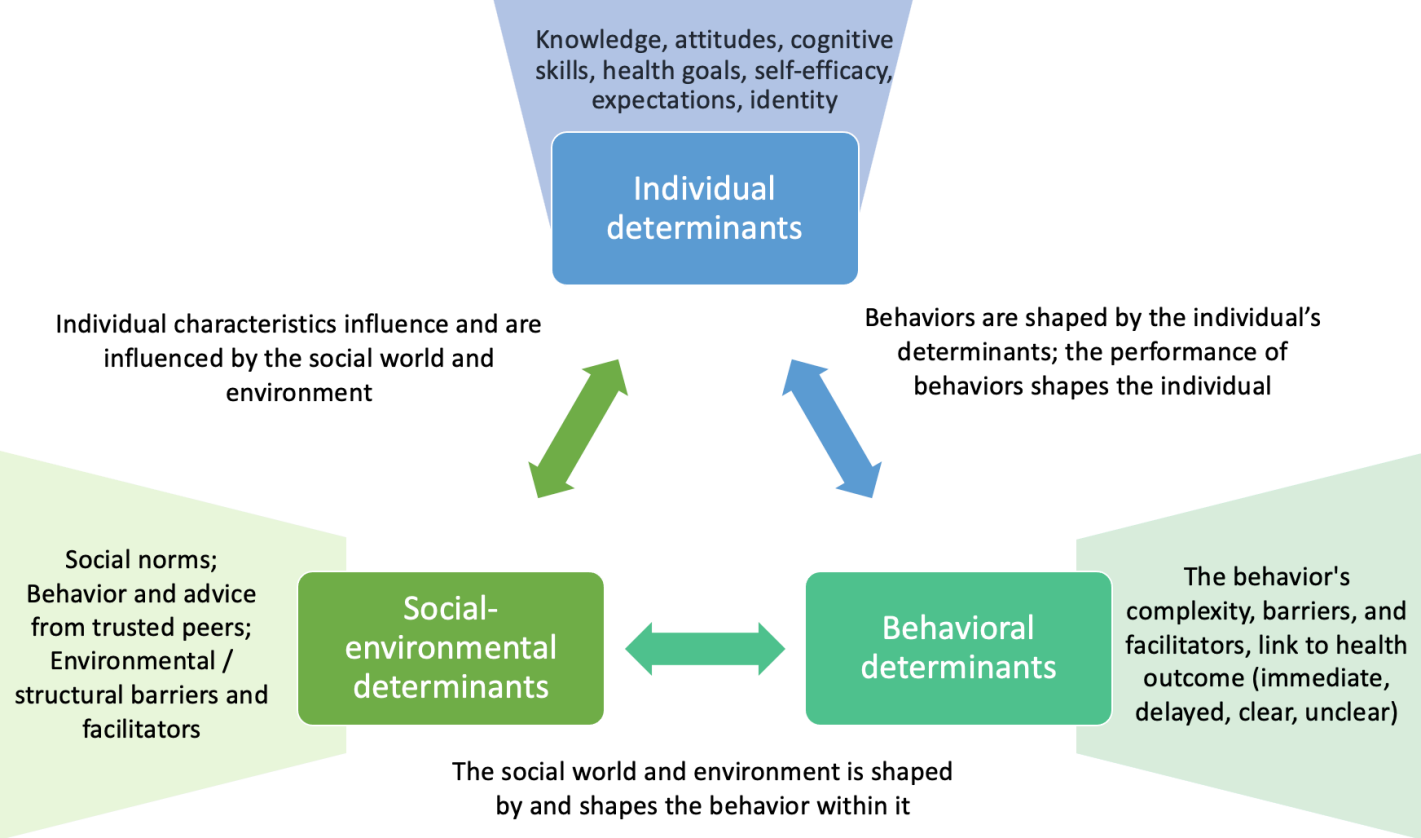
The reciprocal interaction of individual, social–environmental and behavioural determinants.

We draw from Bandura’s theory of reciprocal determinism,^22^ which explains behaviour as being determined by the interaction of individual attributes, the social environment, and the behaviour itself, to examine IYCF and family planning behaviours in relation to Kilkari messages. Bandura defines individual attributes to include one’s knowledge, self-efficacy, expected outcomes, health goals, and concrete plans; we add to this conceptualisation the respondent’s worldview, for example, how she makes sense of the world and the extent to which the behaviour in question resonates with her social identity and understanding of what is logical and true in her world.^23^ One’s environment of social, community, and family norms as well as structural environmental barriers and facilitators interacts with these individual level factors to influence behaviour. Furthermore, the past and ongoing performance of behaviours themselves contribute to determining future behaviour—whether to reinforce or challenge one’s individual attributes and to reinforce or challenge the socio-environmental status quo (figure 1).

## Methods

### Respondents

We interviewed 29 women enrolled in Kilkari, whose mobile numbers showed very high (n=9), high (n=13) and medium (n=7) listenership, as assessed by the percentage of cumulative Kilkari call content that was picked up and allowed to play before being hung up (table 1). We purposively sampled a balance of women who were classified at baseline as phone owners and non-owners (table 1). We did not interview women whose registered mobile numbers rarely or never picked up Kilkari calls because they would be largely unable to discuss their impressions of the programme.

**Table 1:** Respondent profiles

Respondent characteristic	N=29	%
Kilkari listening		
Very high listeners (picked up and played 75% or more of the calls for which the subscribed mobile number was eligible)	9	31
High listeners (picked up and played 50%–74% of the calls for which the subscribed mobile number was eligible)	13	45
Medium listeners (picked up and played 25%–49% of the calls for which the subscribed mobile number was eligible)	7	24
Low listeners (picked up and played 0%–24% of the calls for which the subscribed mobile number was eligible)	0	0
Call cutters	4	14
Kilkari woman’s phone ownership		
Owns her own phone	15	52
Does not own her own phone (shares a phone)	14	48
Family structure		
Lives in a nuclear family	6	21
Lives in an extended family	23	79
Caste		
General	6	21
Other backwards caste	10	34
Scheduled tribe	4	14
Scheduled caste	9	31
Kilkari woman’s literacy		
Cannot read a full sentence	13	45
Can read a full sentence	16	55
Household wealth		
Poor (Q1 and Q2)	15	52
Middle (Q3)	6	21
Wealthier (Q4 and Q5)	8	28

**Figure 2:**
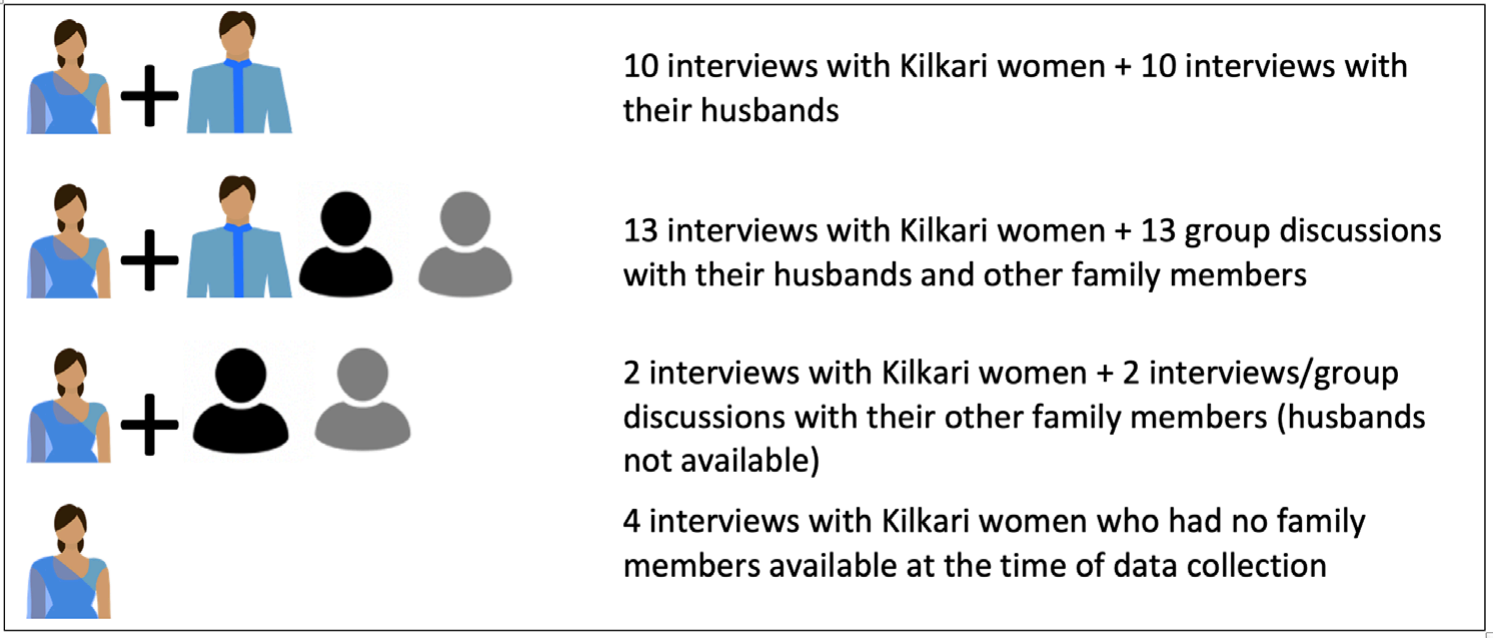
Visual representation of qualitative respondent data set.

We aimed to also conduct a separate interview with each woman’s husband alone or alongside additional household members (table 2, figure 2). We interviewed husbands to understand the extent to which they picked up and listened to Kilkari calls, their reactions to Kilkari content on IYCF and family planning, and the extent to which they engaged in discussion with their wives about these topics. We included other family members and particularly mothers-in-law to understand Kilkari calls in the context of the woman’s broader social world, including what other power brokers in the home thought about Kilkari and about IYCF and family planning.

**Table 2:** Research participants and data collection methodologies

Research participants	N
Kilkari women (mothers of 1-year-old children who were enrolled in Kilkari while pregnant)	29
Husbands of Kilkari women	23
Mothers-in-law of Kilkari women	13
Other family members of Kilkari women	12
**Data collection methodology**	
One-on-one in-depth interviews	
*With Kilkari women*	29
*With either the woman’s husband or extended family*	10
Family group interviews	15

Among our respondents, the mobile numbers registered to Kilkari listened to an average of 33 calls (range: 16–50) and were eligible to receive an average of 52 calls (range: 39–57) based on timing of enrolment and current age of their child. At the time of fieldwork, 22 of the 29 subscribers had been enrolled in Kilkari long enough to receive all family planning and IYCF content (online supplemental file 2).

10.1136/bmjgh-2021-005868.supp2 Supplementary data

### Data collection

Interviews took place in rural Madhya Pradesh in late 2019 and began with a discussion of the mobile phones in the home, including who uses the phones and in what capacity. We then discussed Kilkari, including who listened to the messages, their impressions and recollection of the programme, and why calls were missed or cut. We then asked specifically about IYCF practices and family planning. The sequence of questioning on these topics is summarised in table 3.

**Table 3:** Interview strategy for IYCF and family planning topics

Interview stage	IYCF	Family planning
Stage 1: initial discussion	All interviews and family group discussions: Experience breastfeeding and whether anything other than breast milk was given to the baby in the first 6 months, such as ghutti and water; Time of initiation of complementary feeding; Dietary diversity and thickness of foods, including whether dal ka paani was given and for how long; Frequency of feeding and portion size; Discussions and decision-making on IYCF; Recall of any Kilkari content on IYCF.	Interviews with women and husbands: Use of family planning since the baby’s birth; Desired number of children; Desired spacing between children; Discussions and decision-making related to family planning; Recall of any Kilkari content on family planning. Family group discussions: General feelings about the ideal number of children, space between pregnancies and use of contraceptives.
Stage 2: Kilkari messages played	All interviews and family group discussions: #26 exclusive breastfeeding (no water or ghutti) and #49 thick foods (no dal ka paani).	Interviews with women and husbands: #60 on spacing pregnancies, OR #54 on limiting family size, OR One of the messages on modern methods: #34 IUCD, #55 male sterilisation, #65 OCP, #66 condoms, #68 ECP. Family group discussions: No message on family planning played due to sensitivity.
Stage 3: follow-up discussion	All interviews and family group discussions: What was the message saying? Do you agree? Why did you practice X when Kilkari says to do Y? How do you feel about being told not to give water / ghutti / dal ka paani? What would others think if you stopped giving these items?	Interviews with women and husbands: What was the message saying? Do you agree? How do you feel about being told to have fewer children / space pregnancies / to use this method of contraception? What would others think if you attempted to follow this Kilkari advice? Interviews and family group discussions: Why do some people have more than two children / have only 1-year gap between children / not use modern contraception?

ECP, emergency contraceptive pill; IUCD, intra-uterine contraceptive device; IYCF, infant and young child feeding; IYCF, infant and young child feeding; OCP, oral contraceptive pill.

Kilkari messages that were played (in Hindi) in the interviews are presented below (table 4). Kilkari encouraged exclusive breastfeeding and asked listeners not to give babies water, sweets, or ghutti, a paste that can be made at home or purchased from shops and includes a wide range of potential ingredients such as ground animal bone or antler (barasingha), dates, almonds, cashews, and raisins. The interviewers decided during the interview which Kilkari messages to play for respondents based on the information provided in the initial discussion. They sought to play messages that explored areas of divergence between Kilkari directives and respondent practices.

**Table 4:** Examples of Kilkari messages played during interviews

Topic	Content
#26 Exclusive breastfeeding (no water or ghutti)	Hello Bhaiyya–Bhabhi (brother–sister, referring to the woman and her husband)! How are you? I hope you are well! I am Dr Anita and I am calling from Government of India’s Kilkari Mobile Service. Do you know why the children on television are so healthy and smart? Because they drink only breast milk for the first 6 months, nothing else, not even honey, sugar, ghutti or water. Mother’s milk is the complete diet for babies. Make sure you breastfeed your baby 8–10 times a day. And if the baby demands more, then feed more. This means breastfeed a 6-month-old baby as and when it wants. Always wash hands before putting your child to breast. Remember, breast milk helps build immunity in your child. An exclusively breastfed baby is healthier and its mind and body grow well, even after childhood.
#49 Thick foods (no dal ka paani)	Hello Bhaiyya–Bhabhi! How are you? I hope you are well! I am Dr Anita and I am calling from Government of India’s Kilkari Mobile Service. Your child is now older than 6 months and so must be eating food other than breast milk. Do not feed your child liquid food like daal ka paani. Make sure whatever you feed to your baby is thick in consistency so that it does not flow through your fingers. Your baby will be stronger and more energetic if you feed it thick food. If the food is too watery then your baby may not get adequate nutrition. Remember to wash hands while preparing the food and before feeding the baby. Remember to give more food to the baby during illness—this helps the baby to recover faster.
#34 IUCD	Hello Bhaiyya–Bhabhi! How are you? I hope you are well! I am Dr Anita and I am calling from Government of India’s Kilkari Mobile Service. Are you worried about getting pregnant again or thinking about how to limit your family’s size? Are you wondering about which method will prevent pregnancy right now but will still allow you to have a child later? The IUCD is a simple and effective solution to your worries. There are two kinds of IUCDs—one that lasts for 5 years and one that lasts for 10 years. It can be inserted at a health facility by a trained doctor or a nurse 6 weeks post delivery. And remember you can have it removed whenever you want to have a baby. The IUCD is inserted for free. Talk to your ASHA/auxiliary nurse midwives about choosing an IUCD today. (The following messages are in annexure 1: #54 limiting; #55 male sterilisation; #58 female sterilisation; #60 spacing; #65 OCP; #66 condoms; #68 ECP)

ASHA, accredited social health activist; ECP, emergency contraceptive pill; IUCD, intra-uterine contraceptive device; OCP, oral contraceptive pill.

### Analysis

After each day of data collection, the research team engaged in extensive structured debriefs, guided by the analysis. These debriefs involved filling in a spreadsheet to summarise findings from each interview across our domains of interest (box 1).

Box 1 **Analysis framework**

**General characteristics**

- Family structure, number of children, education, etc.

**Mobile phones**

- Phones in the house/condition.
- Digital literacy situation/what the phones are used for.
- Woman’s access.
- Norms and values around phones (What is ‘good’ use and ‘bad’ use? Spam calls? Would they like to see women have more access?).

**Barriers/facilitators to receiving Kilkari calls**

- How regular is Kilkari? What did you think the first time you got a call? What number does it come from? How do you know if it’s Kilkari?
- Who answers/listens to the calls? Why?
- Why were some calls cut or missed?

**Experience with Kilkari**

- What Kilkari was about, what they remember about it.
- Good things about Kilkari.
- What Kilkari can be improved.
- What do various family members/others think about Kilkari? Any discussions about Kilkari?

**Infant and young child feeding (IYCF) practice**

- Exclusive breastfeeding situation.
- Complementary feeding situation.

**Link between IYCF practice and Kilkari**

- What do respondents remember about Kilkari content on IYCF?
- Any discussions about IYCF after hearing something on Kilkari? Any discussions on IYCF in general?
- Reaction to Kilkari message on exclusive breastfeeding; facilitators and barriers to exclusive breastfeeding.
- Reaction to Kilkari message on complementary feeding; facilitators and barriers to appropriate complementary feeding.

**Family planning practice**

- Family planning (FP) practice since having the baby.
- Family size, desire, reasoning.
- Spacing in practice, desire, reasoning.

**Family planning**

- What did Kilkari say about FP (size, spacing, methods)? Would you prefer to learn about FP from a health worker or Kilkari and why?
- Any discussions about FP after hearing something on Kilkari?
- Barriers to using modern methods/reasons families do not use modern methods.
- Reasons families have more than two children.
- Barriers to 3-year age gap between children.

Audio recordings were transcribed and translated into English and coded using the same framework. We then synthesised findings across codes to identify primary and tertiary themes. A secondary stage of analysis involved re-reading transcript pairs as household case studies (woman and husband or family). Finally, we re-examined the data on respondent interpretation of Kilkari as well as facilitators and barriers to adopting the Kilkari recommended practices through the theoretical framework of reciprocal determinism, focusing on how Kilkari’s recommendations were comprehended and reconciled by listeners in relation to behavioural, personal, and socio-environmental determinants.

### Patient and public involvement

The research participants were not involved in the design or reporting of this study, and the results have not been disseminated to them.

## Findings

### Kilkari listening and general impressions

The woman was the primary listener to the Kilkari calls in only one-third of the households; these were the women who owned their own phones and had access to their phones throughout the day (table 5). For one-third of the families we interviewed, it was the husband who listened to all or most of the Kilkari calls; these were the families where the woman reported sharing a phone. In the remaining one-third of the cases, the woman and her husband each reported listening to some of the calls.

**Table 5:** Who actually listened to Kilkari?

Listener type	Description	Examples
Woman was the listener (WOM_02, WOM_05, WOM_06, WOM_07, WOM_11, WOM_12, WOM_18, WOM_21, WOM_25)	Woman owned her own phone and kept it with her most or all of the time. She reported picking up all or almost all Kilkari calls.	WOM_05 owned her own phone and picked it up almost all the time. When her husband was home when the Kilkari call came in he sometimes listened to it with her. WOM_07 owned the phone and picked it up almost all the time. But her younger sister-in-law picked up the calls sometimes when she heard the phone ring and WOM_07 was not nearby.
Husband was the listener (WOM_09, WOM_13, WOM_14, WOM_16, WOM_19, WOM_22, WOM_24, WOM_26, WOM_29)	Husband owned the phone and listened to all or most the Kilkari calls that were answered on that phone.	WOM_13 owned her own phone at the time of the interview (1-year post partum) but at baseline (while she was pregnant) only her husband had a phone, so his mobile was registered to Kilkari. WOM_26 did not own her own phone. Kilkari calls came in on her husband’s phone, which he kept with him at all times. WOM_26 knew it was a weekly call but had only heard two messages. Other than those two calls, her husband listened and told her that calls came. He said he recorded Kilkari calls and played them for her but she did not appear to have heard these recordings.
Husband was the main listener, woman was an occasional listener (WOM_01, WOM_03, WOM_04, WOM_08, WOM_10, WOM_15, WOM_23, WOM_28)	Husband owned or was the primary user of the phone and picked up Kilkari calls when he could. When he received a call while close to his wife he may give her the phone or play the call on speaker.	WOM_01 did not have her own phone but her husband left his phone with her so that they could keep in touch while he stayed in the city to receive medical treatment. Before he left he received most calls. While he temporarily moved away she received the calls. At one point she spent a month at her natal home and the phone remained with her husband. WOM_03 owned the phone (it was given to her by her brother) but when her husband went out he took it with him. WOM_03 and HUS_03 both reported that they were the ones who picked up ‘most’ of the Kilkari calls. If the husband picked it up and was home with his wife, he handed it to her. They both estimated they heard one message a month. WOM_04’s phone broke 3 months ago so she did not have her own phone when we interviewed her. Her SIM was in her husband’s phone. She thought the calls came once or twice a month and estimated that she had heard four to six Kilkari messages, in total. Her husband also said Kilkari came every 2 weeks. When the call came in to the husband’s phone while he was home with his wife, he handed the phone to her or listened with her. WOM_10’s extended family shared one phone, which stayed at home while the husband went out to work. Both WOM_10 and HUS_10 reported that the other person received most of the calls and had the phone most of the time. WOM_23 owned her own phone but everyone in her extended family picked it up when it rang. Her husband, sister-in-law and mother-in-law have all listened to some Kilkari messages. Her husband estimated that he heard around six Kilkari messages. WOM_28 did not have her own phone. She had no sense of how frequent Kilkari calls were because they came into her husband’s phone Despite this, she estimated that she had heard six or seven calls because her husband played Kilkari on speakerphone to her sometimes.
There was also one special listenership profile: Unexpected person was the high or medium listener: WOM_17 did not own her own phone; her father-in-law’s phone has been enrolled into Kilkari. He lived in another house in the adjoining compound and was listening to the messages but did not tell anyone about them.		

Respondents said that the most common reason they did not pick up incoming Kilkari calls, or ‘cut’ the call quickly, was because they were busy with work—whether domestic work for women or work outside the home for men. Additional reasons for missing calls included: SIMs being deactivated temporarily because the phone was not recharged with credit or because the SIM’s registration was incomplete; phones switched off while being repaired or because the battery has drained; women travelling to their natal home and leaving their phone behind; women feeling unwell and thus not picking up incoming calls; phone speaker broken, and network trouble. Additional reasons for cutting calls included feeling the messages were too long, difficult to understand, covered content they already knew, or were not important.

We asked whether respondents ever saw that a Kilkari call was incoming and decided not to pick it up because they did not want to listen to Kilkari. Respondents rejected this suggestion, saying that they picked up whenever they could. Only a few respondents recognised that a Kilkari call was incoming by the number displayed on their phone (FAM_07, woman; HUS_23, both; HUS_26, husband); most realised it was a Kilkari call once they picked up and heard the introductory message and song.

#### Overall impression of the programme positive but recall was vague

Respondents who recalled receiving Kilkari messages were positive but not effusive about the service, describing the messages as ‘good’, ‘useful’, easy to comprehend, and convenient to receive.

She speaks well, in the language of common people. (WOM_01, both listened)

If someone will come [in person] then they will tell different things. […] Over the phone they will tell one thing only, so it is better. It is convenient. You get the message while sitting at home. (HUS_19, husband listened)

Families that were well served by government health and nutrition service said that the content echoed what they were told by their anganwadi worker and ASHA (‘I haven't got any new information.[…] Yes, it was told in the anganwadi also.’ WOM_04, both listen), which was the intent of the programme. For the families that felt underserved by FLHWs, Kilkari’s value was linked to their otherwise low access to advice and information from healthcare workers. Respondents particularly noted that frontline workers were not a good source of information about family planning, and valued receiving information on this topic through the phone: health workers were widely reported to not talk about family planning, and male respondents said they were too ‘shy’ to talk to female health workers about this topic, were not home when the health workers came or did not trust the health workers (HUS_05, HUS_26, HUS_29, HUS_18).

I like the calls more. […] ASHA didn’t tell much. (WOM_28, both listen).

It is a little difficult to ask. It is easier on the phone because it just comes. We don't like to ask. (HUS_26, husband listens)

Despite these benefits to phone-based messages, two women noted that although Kilkari was filling a gap it would be preferable if FLHW themselves started doing a better job at delivering this information. Several male respondents said that they would have liked to be able to ask questions, whether by phone or through face-to-face interaction with FLHWs (although there are no FLHWs for men).

If she [ASHA] comes and tells [about family planning] it'll be even better [than hearing on Kilkari]. (HUS_23)

Furthermore, although respondents said that the calls were easy to understand, probing on specific vocabulary words used in the calls and discussions after playing the call content during the interviews revealed that women in particular struggled to comprehend some terms and concepts being conveyed (WOM_07, WOM_22, WOM_23, WOM_25, WOM_26).

R: Yes, I can understand. The language used is similar to the one we use to converse. So there are no problems faced in understanding their messages.

I: Are there any instances in which you have failed to understand some complicated words?

R: No.

I: If I say pariwar niyojan [family planning] what do you understand by that?

R: [Long pause]

I: What about garbh nirodhak [contraception]?

R: I cannot remember. (WOM_07, woman listened)

Several dialects are spoken in Madhya Pradesh, including in the districts where the qualitative research was conducted. This is a challenge facing digital direct-to-beneficiary interventions that aim to achieve national scale in India, where 270 official mother tongues and hundreds more dialects are spoken.^24^ It is unclear whether the comprehension issues revealed by the qualitative research were due to low understanding of Hindi in general, or difficulty understanding the technical words chosen, or a combination of both.

Each message began with a statement that Kilkari is from the Government of India; however most respondents did not realise that Kilkari was a government service and appeared not to have considered the source of the calls prior to being asked about this during the interview. Nonetheless, most respondents said they trusted the content, and grounded this trust in their perception that the call content was clearly for the betterment of mother and child.

[I believe her—Dr. Anita—] Because she tells good things to take care of babies like what to feed, etc. (WOM_04, both listen)

Before playing any Kilkari content during the interviews, we asked the respondents what they remembered. Many had a vague recall of the call content, explaining that they did not pay close attention, did not hear many calls, or could not recall what was said.

Sometimes I have missed it and might not have paid that much attention to all the messages. I never thought that some interviewer would come later and ask me if I had listened to all the messages. (WOM_07, woman listened)

First time they said Namaste, and asked me to take care of the baby, take care of the mother, take care of the diet, medicines for the baby. […] I don’t remember anything as such. I don’t remember […] I am busy with chores. (WOM_28, both husband and wife listened).

Among those who could recall specific content areas, respondents said Kilkari was about child health, particularly to immunise and to feed the child home cooked food, fruit, green vegetables, and thick food, and to start complementary feeding at 6 months. Only two respondents spontaneously mentioned content on family planning, with WOM_03 (both listened) noting ‘they told about condoms recently’ and WOM_04 (both listened) recalling being told about a pill and condoms.

### To what extent do respondents communicate about Kilkari and Kilkari topics?

Although husbands and wives often reported telling one another that a Kilkari call came, only two couples said that they spoke about call contents. In one case (WOM_16), the dialogue was a one-time occurrence in which the husband explained the Kilkari call about female sterilisation to his wife after they listened to the calls together on speakerphone. In the other case (WOM_13, husband listened), the husband and wife both said that the husband regularly relayed Kilkari content to his wife. Husbands and wives who listened to some Kilkari calls together on speakerphone still reported that they did not discuss the call when it was over (WOM_28, HUS_04).

R: Sometimes he tells that a call had come from Kilkari. […]

I: Does he tell what they told him?

R: No, he doesn’t tell that. […] When I ask him [about the call], he says, ‘ Nothing, it was just like that’. […] He is not that interested. […] Since he is mostly driving, he doesn’t get the time. (WOM_28, both listened)

Husbands explained that if they heard a Kilkari call while out working, they often forgot about it by the time they came home and did not consider the content to be new or important enough to share with their wives.

R: My attention went a little here and there. So I forgot. […] So far whatever times I heard it’s just that same thing. (HUS_23, both listened)

Discussion among female family members was occasionally reported. In some families, the woman’s mother-in-law and sisters-in-law knew about Kilkari and reported discussing Kilkari content. However, in most families, no one beyond the woman and her husband knew about Kilkari and listeners reported not discussing Kilkari with their family or anyone else.

R: [I don’t tell my mother-in-law because] she may find it non-sensical.

I: Ok. Will she believe these things if she listens to them?

R: I don’t think so. […] She [my MIL] may think that she [Dr Anita] is showing off as a doctor. (WOM_29, husband listened)

### How did respondents interpret and process Kilkari’s specific content?

**Figure 3:**
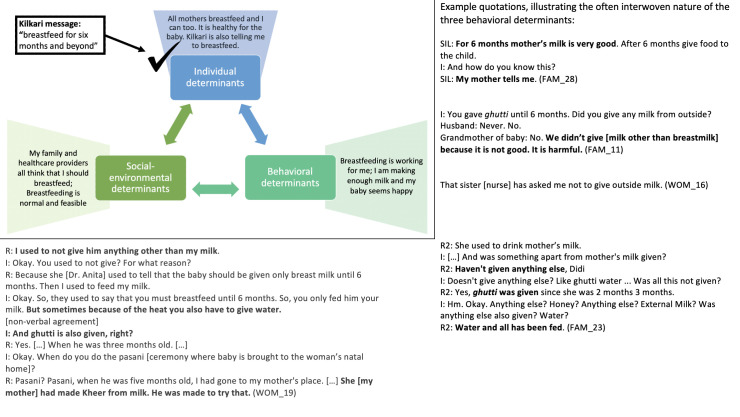
How individual, socio-environmental and behavioural factors interact to support the persistence of breastfeeding, a Kilkari recommended behaviour.

**Figure 4:**
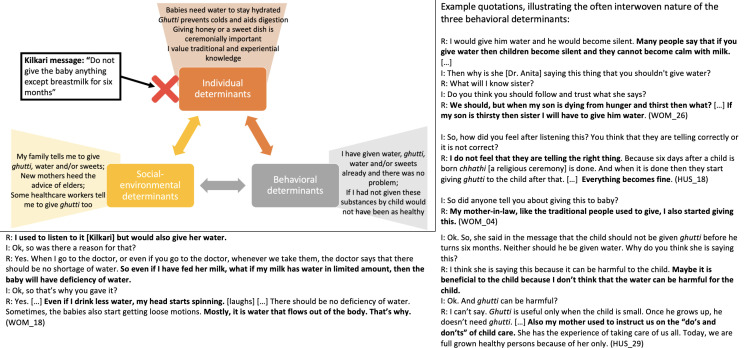
How individual, socio-environmental and behavioural factors interact to support the persistence of non-exclusive breastfeeding, a Kilkari contraindicated behaviour.

**Figure 5:**
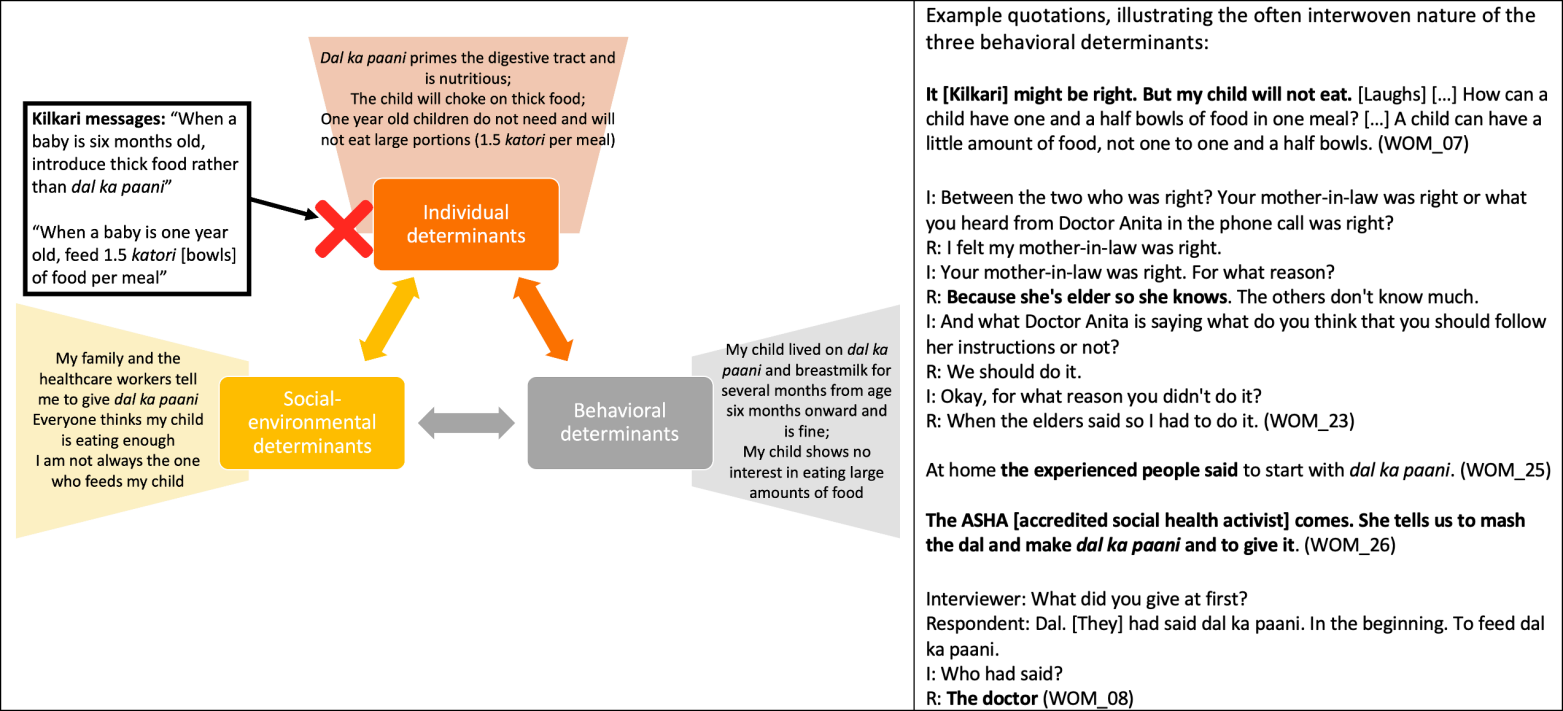
How individual, socio-environmental and behavioural factors interact to support the persistence of giving dal ka paani and small portions of food during complementary feeding, which are Kilkari contraindicated behaviours.

**Figure 6:**
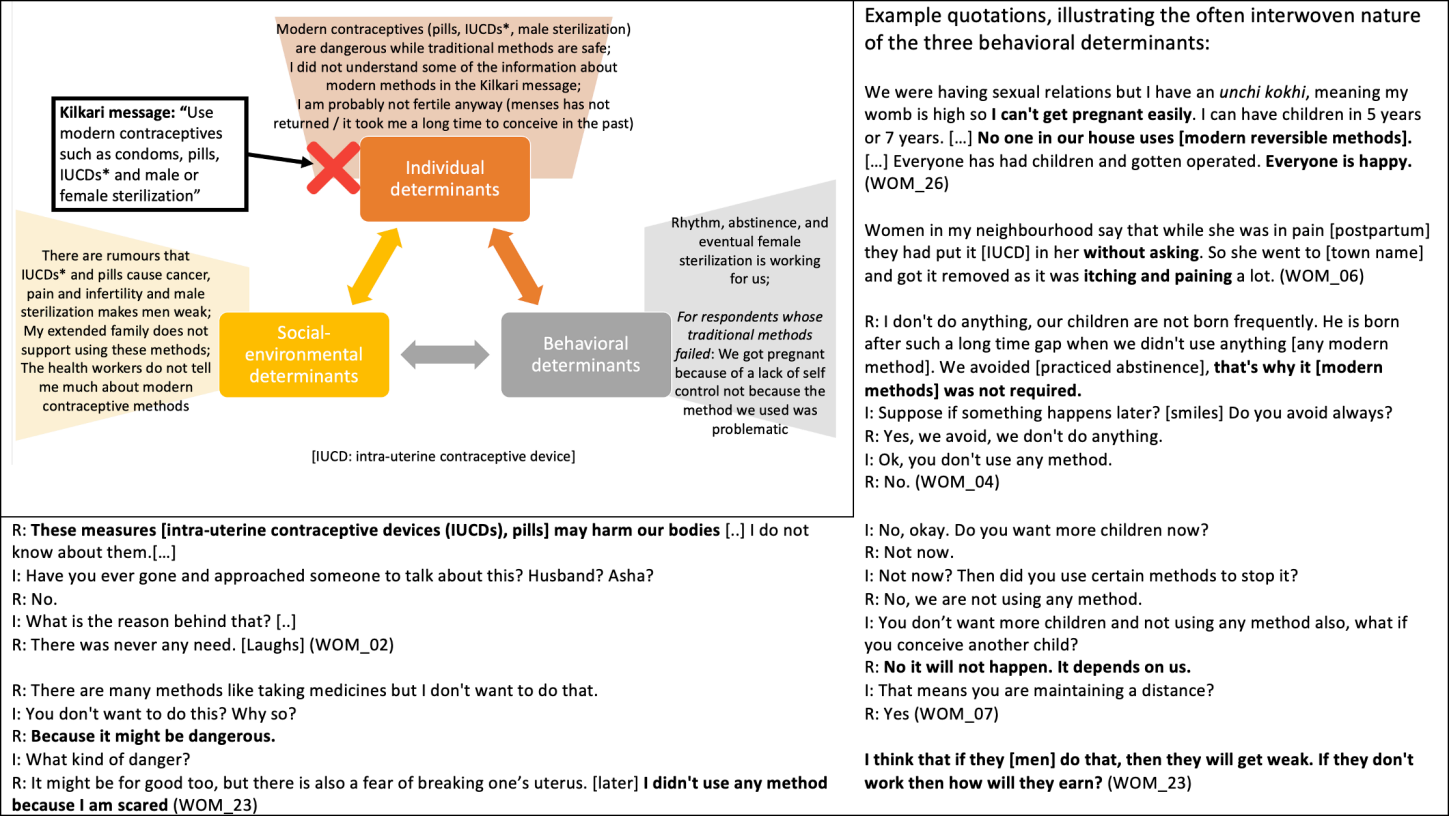
How individual, socio-environmental and behavioural factors interact to support the avoidance of modern reversible family planning and male sterilisation.

**Figure 7:**
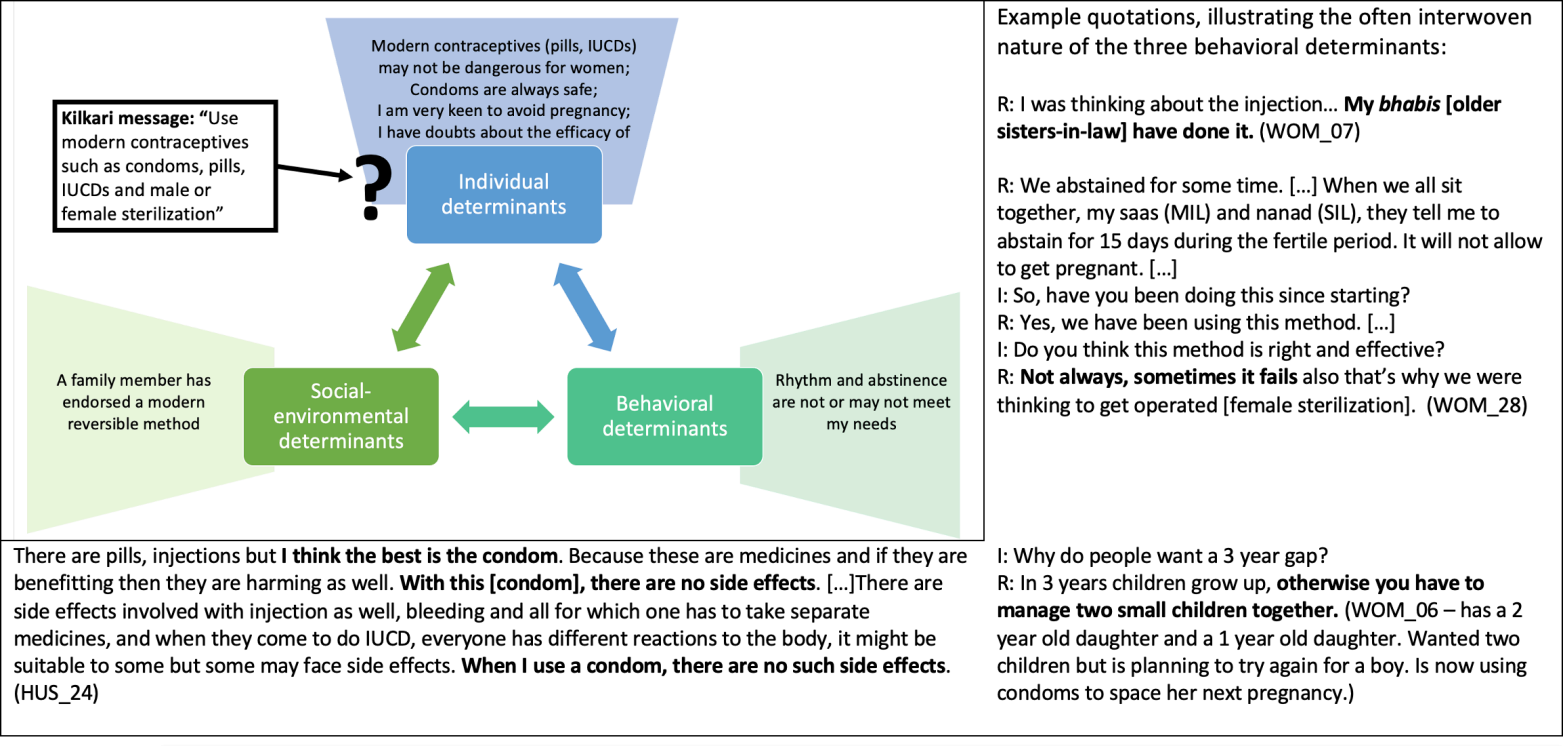
How individual, socio-environmental and behavioural factors interact to potentially enable Kilkari to influence the use of modern reversible family planning.

Listeners retained and appreciated the aspects of Kilkari calls that resonated with existing practices, social norms and personal worldviews but overlooked and de-emphasised Kilkari calls that conflicted with these behavioural determinants (table 6, figures 3–7).

**Table 6:** Kilkari content that was retained versus content that was overlooked

Content that was retained	Content that was overlooked and de-emphasised
Breastfeed for 6 months and beyond. Feed children over 6 months homecooked food. Maintain hygiene. Have a small family.	Do not give children under 6 months any water, sweets or ghutti (herbal paste). Initiate complementary feeding with thick foods not dal ka paani (the water in which lentils are cooked). One-year-old children should be fed 1.5 katori (small bowl) of food at each meal. Modern contraception (specifically pills, intra-uterine contraceptive devices, and male sterilisation) is safe and effective.

Kilkari content that was retained and appreciated was that which resonated with the individual as good practice. Some of this resonance was facilitated by the imprecision of either the message or the individual’s interpretation. For instance, ‘small family’ meant having two children to some respondents, but meant three or four children to others. Many contextualised the correct minimum number of children within the framework of needing at least one son.

I: According to you, there should be three kids. Why 3?

R: Three because I already had two girls and I wanted a boy. At first, I thought if I will have a boy and a girl, I will get myself operated [sterilised]. But when I had two girls, I thought of trying again for a boy. (WOM_28)

The social environment (frontline workers, family members, and the broader community) reinforced the high-resonance messages. Furthermore, respondents felt that they were practicing or planning to practice these behaviours already and interpreted these messages as encouragement to continue as planned.

While respondents reflected on Kilkari as providing good and trustworthy advice that they agreed with, they also reported numerous contraindicated behaviours—seemingly without considering that their practices were contrary to Kilkari’s recommendations. For example, Kilkari’s encouragement that women breastfeed *exclusively* was widely interpreted by listeners to be encouragement that women breastfeed *primarily* (figure 3); thus women were confident that they were adhering to Kilkari’s recommendations while also reporting that they gave water, ghutti, and sweets.

When probed, some respondents said that they had not heard anything specific in Kilkari about the topics that conflicted with their practice, such as giving ghutti and dal ka paani, even when they had listened to the call containing that content. Others misremembered Kilkari as endorsing practices that Kilkari actually discouraged or were unsure whether Kilkari promoted or discouraged these practices. For example, HUS_29 reported that Dr Anita encouraged them to give dal ka paani, explaining that he did not remember exactly what was said but ‘there was some reference about it in the message [so] I used to feed it to the child’. Immediately after being played the Kilkari call that says not to give ghutti, MIL_23 recalled that ‘the doctor’ [Dr Anita] said that ghutti should be given, WOM_29 could not recall what the doctor said about water and ghutti and WOM_19 initially recalled that Dr Anita said not to give ghutti but minutes later adjusted her recollection and said that Kilkari encouraged giving ghutti.

Further questioning, and repeating the low-resonance Kilkari calls during the interviews, eventually resulted in some respondents identifying and discussing a conflict between Kilkari’s recommendations and their practices. Respondents drew from the three core determinants of behaviour in Bandura’s model—personal attributes and worldviews, the perceived ongoing success of their existing practices, and socio-environmental factors—to explain why their contraindicated behaviours were correct and resistant to change in light of Kilkari’s content. These determinants made non-exclusive breastfeeding (figure 4) and insufficient complementary feeding (figure 5) strongly entrenched and resistant to Kilkari’s directives. They highlight the limits of digital direct-to-beneficiary behaviour change communication interventions, which were not designed to work in isolation—but to reinforce the efforts of well trained, adequately equipped, FLHWs to change social norms.

Kilkari’s calls promoting modern contraceptives, particularly pills, injectables, intrauterine devices and male sterilisation, were also largely rebuffed by women respondents (figure 6)—however there were some respondents who showed dissatisfaction with the use of traditional methods, noted socio-environmental support for trying modern methods, and articulated an interest in more information about them (figure 7). These factors may indicate mechanisms through which Kilkari achieved a statistically significant increase in the use of modern reversible methods.

The determinants that drove contraindicated behaviour showcased interaction between deeply held personal beliefs and attitudes that the behaviour was correct, the self-reinforcing experiential ‘evidence’ that the behaviour was succeeding in contributing to good health, avoiding sickness or enabling reproductive goals, and strong social norms to continue in the traditional manner.

Existing practices were self-reinforcing and were interpreted as further evidence that the status quo was acceptable. Few respondents identified problems with their existing practice, nor did they link negative occurrences to Kilkari’s contraindicated behaviours. Diarrhoea and respiratory issues in babies were commonly reported during the interviews but non-exclusive breastfeeding (which we identified in all but one of the families interviewed) was seen as a contributing factor to the child’s recovery or a reason the child did not experience additional or more severe illness. Every female respondent reported giving their child dal ka paani, often as the child’s primary food alongside breast milk for several months upon the initiation of complementary feeding (eg, WOM_06 gave her daughter only dal ka paani, water and breast milk from 6 to 11 months). All respondents reported that their 1-year-old child ate just a few spoonfuls of food two to four times per day (much less than the Kilkari-recommended portions and frequency). Yet no one expressed concern that their child was under-nourished or had insufficient appetite, even the parents of a child who had been referred to a supplementary feeding centre due to being underweight (FAM_05).

While almost all couples interviewed wanted to delay or avoid pregnancy, only one woman had been sterilised (WOM_08) and only four couples were using condoms (WOM_06, WOM_07, WOM_09 and WOM_11). Respondents explained the persistence of traditional methods in terms of avoiding greater harm caused invasive modern methods (eg, pain, bleeding and weakness linked to pills, injectables, intra-uterine contraceptive devices and male sterilisation), satisfaction with traditional methods and—for some but not all respondents—low access to and awareness of modern contraceptives (figure 6). Many also mentioned belief that they were not fertile because their menses had not yet started 1-year post partum or because of prior experiences of infertility. Nonetheless, some recognised that traditional methods (rhythm and abstinence) were fallible and articulated some social norms in support of trying reversible modern methods (figure 7). In addition, while norms against injectables, pills, injectables, intrauterine devices, and male sterilisation were grounded in fear of negative health impacts, the norms against condoms appeared less substantive: some respondents said they simply did not need them or know about them, and others felt shy or ‘disgusted’ (WOM_18) by them. The benign nature of condoms may explain why condom use in particular increased among Kilkari listeners, while the other methods were more resistant to change.

Most respondents spoke of the important knowledge held by elders. Women said they had to follow their mother-in-law’s directives but also believed that these behaviours were correct. For example, WOM_23 said that she told her mother-in-law that Kilkari recommended not giving ghutti but that her mother-in-law justified continuing to give ghutti and that she had to do what she is told by her elders (‘I didn't follow her [Dr Anita] because whatever the elders will say we follow that only.’ (WOM_23)). However, WOM_23 also endorsed the principle of following one’s elders by explaining that elders are knowledgeable and that ghutti is beneficial to the child’s health. Later, WOM_23 was asked whether she would wait 6 months before giving ghutti to her next baby. She first replied confidently that she would do so, but seconds later said that ‘whatever the older people will say we will do that’. When pushed on why she would follow her elders’ advice and not Dr Anita’s she ultimately explained ‘I don't know why she [Dr. Anita] doesn't want to give ghutti to the child or what reason is there; I don't know about that’ (WOM_23).

## Discussion

This qualitative study explored issues of Kilkari reach and resonance in rural Madhya Pradesh. In terms of reach, women who owned their own phone and kept the phone with them throughout the day received Kilkari; when women shared a phone with their husbands the husbands were often the listeners. Discussion of Kilkari among household members was rare. In terms of call content resonance, we found that although listeners said they heeded and trusted Kilkari’s advice, they primarily absorbed and recollected calls that aligned with existing practices, social norms, and personal worldviews. Messages that conflicted with these three core behavioural determinants were often overlooked and de-emphasised, including calls promoting exclusive breastfeeding, initiating complementary feeding with thick foods, giving sufficient amounts of food, and using modern reversible contraceptives, particularly pills, injectables, intrauterine devices, and male sterilisation.

The quantitative Kilkari RCT results indicate that exposure to Kilkari had a significant impact on the adoption of modern contraceptive methods.^21^ However this qualitative study did not identify any clear linkage between receiving Kilkari calls and changing one’s family planning practice, which is perhaps unsurprising given that the difference in uptake of modern contraceptive methods between exposed and unexposed was 8%. The qualitative research does, however, support the quantitative finding that Kilkari did not have an impact on IYCF practices and provides some evidence of an unmet need for family planning. We now draw from our qualitative research to hypothesise why the quantitative RCT found that exposure to Kilkari was associated with a change in family planning and not in IYCF practice.

First, while respondents in the qualitative study expressed satisfaction with the status quo around breastfeeding and complementary feeding practice, some couples indicated a degree of dissatisfaction with traditional family planning practices. Respondents did not link childhood illness to suboptimal IYCF, and in fact considered contraindicated feeding behaviours (specifically giving ghutti and water before 6 months and focusing on the insufficiently nutrient-dense dal ka paani for the first period of complementary feeding) to be health promoting. No one expressed concern about low appetite or poor growth among their children, echoing findings elsewhere that parents have low recognition of underweight status among their children.^25 26^ This points to insufficient counselling by FLHWs on infant nutrition and gaps in frontline worker knowledge and capacity.^27^ While most qualitative respondents were similarly satisfied with their traditional approaches to family planning, several did acknowledge the fallibility of these methods and almost all wanted to space pregnancies and have just two or three children provided they had at least one son. A single digital direct-to-beneficiary behaviour change communication intervention is very unlikely to change deeply entrenched social and behavioural norms, such as son preference and child feeding behaviours. According to the programme’s theory of change, Kilkari was supposed to reinforce face-to-face communication by well trained, well equipped FLHWs.^28^ Nonetheless, the change that Kilkari did effect could be deepened by more frequently repeating the calls on misunderstood or overlooked concepts, such as dal ka paani, exclusive breastfeeding and contraceptive options, and by engaging with common reasons for the persistence of contraindicated behaviours.

Second, when husbands listened to IYCF messages, these messages would have low capacity to impact IYCF behaviour because men were minimally involved in child feeding and did not share information from Kilkari with their spouses. However, when husbands listened to family planning calls, these calls could have equal or even heightened capacity to impact family planning practice, since husbands were equal or greater stakeholders in decision-making about contraception. Future programmes can consider designing specific outgoing messaging programmes to target husbands, who are easier to reach by mobile.

Third, and closely linked to the previous two points, respondents articulated a desire for reliable information about family planning. Most women reported learning about breastfeeding and complementary feeding from their FLHWs and elders. However, respondents said that they learnt very little about contraception from FLHWs, that FLHWs promoted female sterilisation and little else, and that inter-family communication about contraception remained focused on traditional methods and female sterilisation. Husbands did not have access to male health workers who they could speak to about contraception. There is clearly a need for FLHWs to receive more training, not just in modern contraceptive methods, but to build their confidence in discussing this taboo topic with families. There also seems to be a need, which Kilkari has begun to meet, for men to receive additional, targeted information about modern contraceptive practices.

## Conclusion

Although the overall Kilkari impact evaluation has demonstrated that a digital direct-to-beneficiary programme can increase the adoption of modern contraceptive methods, it is likely that the impact of the intervention would be broader and deeper if complemented by more effective face-to-face counselling, bolstered by job aids that support facilitated communication. Moreover, given that Kilkari calls were often answered by husbands, it is worth considering whether the impact of Kilkari on modern contraceptive use could be increased by developing digital communications that just target men. Women’s low phone access meant that they missed many calls. Moreover, Kilkari’s recommendations engage with social and environmental determinants, individual attributes, and ongoing behavioural feedback that is frequently working against the absorption of messages and adherence to recommended practices. Digital direct-to-beneficiary behaviour change communication is best understood as part of broader efforts to change social norms and behaviour, which should including mass media communication and face-to-face interaction with health workers or other behaviour change agents, as well as engagement with social and environmental behavioural determinants.

## Data Availability

Data are available upon request. Data for this study consist of qualitative interview transcripts. Uploading all transcripts for open availability would compromise our ability to fully mask participant details. However, we are happy to share anonymised portions of these transcripts upon reasonable request.

## Footnotes

Women who do not want any more children or want to delay the birth of the next child for at least 2 years and yet are not using any contraceptive method.
