# Supplementary material for: Another voice in the crowd: the challenge of changing family planning and child feeding practices through mHealth messaging in rural central India
Source: BMJ Glob Health. 2021 Jul 26;6(Suppl 5):e005868. doi: 10.1136/bmjgh-2021-005868 (PMC8327813; doi:10.1136/bmjgh-2021-005868)
Supplement: Supplementary data [file bmjgh-2021-005868supp001.pdf]

Supplementary file 1. Kilkari messages

Week	Topic(s)	Message (English)
**1**	Welcome	Hello, I am Dr Anita and I am calling from Government of India's Kilkari mobile service. This service is free and you will not be charged for it at all. You are receiving this call because you registered your pregnancy with the ASHA/ANM. From now on I will call you once every week and I will tell you about some important things that you can do easily. These tips will ensure the health of mother and child. So remember to listen to my message every week - let's work together to ensure the best future for your child.
**2 - 16**	Multiple	[Messages 2 to 16 are sent out during the second and third trimesters. Our respondents generally started receiving Kilkari messages in their last month of pregnancy, corresponding to Kilkari message 17 or later.]
**17**	Immediate breastfeeding	It is very important to breastfeed your child immediately after delivery. The first thick yellow breastmilk is like nectar for your baby. It helps protect your baby from diseases. It is like the first dose of immunization for your baby. So don't delay breastfeeding the baby and ensure that your baby is breastfed within the first hour after birth. Also remember - breast milk has everything your baby needs including sufficient water. Your baby does not need any food or liquid other than breastmilk and feeding or making the baby drink other things can make the baby sick so avoid them completely.
**18**	Immunization	Hello expectant parents - it's almost time for your baby to arrive. The biggest responsibility you have as parents is to ensure that your child is immunised. There are some vaccinations that need to be administered as soon as the baby is born. I'm sure you've arranged for the delivery to be in the hospital, in which case the doctor/nurse will give these to your newborn after delivery. If for some reason you are unable to go to the hospital for delivery, make sure you remember to take your child to a nearby health centre immediately after birth and get your child immunised. This will save your child from life-threatening diseases.
**19**	Postpartum checkup	After delivery, post natal checkups for the mother and baby ensure that both mother and baby are well and in case there is a health complication you will be able to get medical help in time. The ASHA is trained to check the mother and baby and is supposed to visit you three times within the first week of delivery to check on the mother and baby. She is then supposed to visit you four more times in the next six weeks. Make sure that the doctor/ANM/ASHA comes to check mother and baby after delivery. If you have any problems, call the free ambulance service and visit the hospital.
**20**	Postpartum IUCD, female sterilization	The time right after delivery is a good time to adopt a method of family planning. After the delivery, a woman has two family planning options: first, a woman can opt for either a PPIUCD insertion or if their family size is complete she can undergo second, female sterilization. The PPIUCD can be inserted within 48 hours after delivery and can be removed when she wants to have another child. Postpartum sterilization can be performed within 7 days of delivery which is a permanent method and can be adopted by the woman when her family is complete and she doesn't want any more children. These services are available free of cost at all government health facilities. For more information contact your ASHA, ANM or doctor.
**21**	Thermal Care	The baby is used to the warmth of the mother's stomach and you must make sure to keep it warm after it is born. If the baby does not get the warmth that it received while in the mother's womb, after birth, it can catch a cold. That's why it is important that the mother provide the baby with ample body heat. Remember, you must not bathe the baby for at least 48 hours after birth. Keep the baby properly wrapped in clothes, and make especially sure that its head and feet are covered. If the baby's skin feels excessively warm or cold, contact the ASHA or ANM immediately or visit a nearby health centre.

Scott K, *et al. BMJ Global Health* 2021; 6:e005868. doi: 10.1136/bmjgh-2021-005868

**22**	Cord care	If the umbilical cord is not looked after properly, it could get infected. This infection could spread to the baby's body and become life-threatening. Keep these three things in mind - first, do not apply anything on the cord, as it will dry and fall off on its own; second, keep the cord clean and dry- if the cord turns red, starts oozing or shows any other signs of trouble, contact theASHA/ANM/nearest health center immediately; third, you need a new soap, new blade and new thread to cut the cord. If the delivery takes place in a hospital, the doctor or nurse will ensure that everything is new. immediately.
**23**	Immediate breastfeeding	It is very important to breastfeed your child immediately after delivery. The mother's first thick yellow breastmilk is like nectar to the child and protects the child from illnesses. It is like the first dose of immunization for your baby. So don't delay breastfeeding the baby and breastfeed the baby within one hour of birth. Also remember - breast milk is the complete diet for you baby. It has everything your baby needs including sufficient water. So there is no need to feed your baby any other food or liquids. Feeding or making the baby drink other things can make the baby sick so avoid them completely.
**24**	IUCD	You will soon be welcoming a new member of the family. This is the right time to make decisions regarding family planning. Because you can concieve again, soon after delivery, even if you are breastfeeding the baby. So how to protect yourself from an unwanted pregnancy? You can get an IUCD inserted for free at a government health facility which can protect you from unwanted pregnancies for a long time. It can be inserted within 48 hours after delivery while you are at the hospital so you wont have to make another trip. If you can not get it inserted within 48 hours then you will have to wait for 6 weeks before having it inserted. And remember you can have it removed whenever you want to have a baby. It's very simple and done for free at the government hospital. Contact your ASHA/ANM immediately for more information.
**25**	Postpartum checkup WASH	After birth, when you look at the baby, it seems like there are no problems now. But remember that the first few days after birth are free of danger for both the mother and the child. After delivery, post natal checkups for the mother and baby ensure that both mother and baby are well and in case there is a health complication you will be able to get medical help in time. Remember, the ASHA is trained to check the mother and baby and should have visited you three times within the first week of delivery. She is then supposed to visit you four more times in the next six weeks to ensure that both the mother and the child are well. Make sure that the doctor/ANM/ASHA comes to check mother and baby after delivery. If there is still some problem, call the free ambulance service and visit the hospital.
**26**	Exclusive breastfeeding	Do you know why the children on TV are so healthy and smart? Because they drink only breast milk for the first six months, nothing else, not even honey, sugar, ghutti or water. Mother's milk is the complete diet for babies. Make sure you breastfeed your baby 8-10 times a day. And if the baby demands more, then feed more. This means breastfeed a 6 month old baby as and when it wants. Always wash hands before putting your child to breast. Remember, breast milk helps build immunity in your child. An exclusively breastfed baby is healthier and its mind and body grow well, even after childhood.
**27**	Child danger signs	A newborn child is fragile and it is easy for it to catch an infection. Listen carefully to some initial symptoms which indicate that the child is dangerously ill - the baby is having difficulty breathing, loses interest in breastfeeding, or is excessively cold or warm to touch. These symptoms might be indicative of a serious illness. Make sure you contact the ASHA/ANM/nearby health centre immediately if these symptoms appear. Don't worry - treatment is free, and going to the hospital can help save the baby's life. Make sure you don't delay seeking medical help.

**28**	Immunization	Immunisation is the best way to ensure your child's health, and it's time for your baby to get its next vaccination. Your baby is due for a vaccination at the age of one and a half months. The ANM visits your village every month to administer vaccinations. Take your child to the nearest health centre. And remember to carry your MCP card - Mother and child protection card with you. Don't forget to get your child immunised when it becomes a month and a half old, and if for some reason you missed the previous vaccination, make sure your child receives that too. Talk to the ASHA/ANM for more information about this. Vaccination protects your child from illnesses, and being careless about it means placing your child in danger.
**29**	MPA injection	Today, I will tell you about the benefits of FP methods. Everyone around you is starting to use these methods. When are you planning to adopt them? There are several methods of birth control available now. One of the new methods is easy and temporary - MPA injections. It is completely safe and effective; it saves time and money, giving you a better future. One injection prevents pregnancy for 3 months and has no effect on breastfeeding. With Doctor's advice and a full check up, any woman can take MPA injection 6 weeks post delivery. You will also get an MPA card that tracks your next MPA injection date and reminds you to get the injection. If you have any problem with the MPA injection talk to your ASHA or health center. Times are changing, spacing between kids will make for a small family happy family. Speak to your ASHA/ANM/Doctor so you can make an informed decision about which family planning method to use.
**30**	Free healthcare for child	Children under 1 year of age are prone to illness, and this can be worrisome. Make sure you avail of the benefits of the Janani Shishu Suraksha Karyakram (JSSK). Under the JSSK, if your child under 1 yr of age falls sick, he/she will be provided hospital stay, treatment, drugs and transport to and from the facility for free at any government health facility. If needed, he/she will also get free blood and referral transport facilities. Ask your ASHA/ANM about the JSSK.
**31**	Exclusive breastfeeding	You already know that you should exclusively breastfeed your baby for 6 months. This is very beneficial for the development of the baby's body and mind. Initially, mothers do face problems with breastfeeding, such as the child being unable to draw the milk, pain in the chest or being unable to feed the child milk. Don't worry if this happens, just contact the ASHA/ANM immediately and ask her what the right way to breastfeed is. Don't start feeding the baby using feeding bottle/nipple and do not give the child any other food or liquids. So, if you're having difficulties, get help from your ASHA/ANM/nearby health centre today.
**32**	Immunization	I know you care for your child, and so you make sure he/she gets timely vaccinations, so that he/she is protected from life-threatening diseases. Your child is about to turn 2 and a half months old, which means that it's time for the next immunisation dose. I am telling right now so you don't forget. Make sure you go for vaccination on the immunisation day at your nearest health facility. Make sure you carry your MCP card- mother and child protection card, along with you. If for some reason you forgot the last vaccination, make sure that your child receives that too. Ask your ASHA/ANM about this.
**33**	Immunization	Tuberculosis, Poliomyelitis, Hepatitis B, Diphtheria, Whooping Cough, Tetanus, Measles, Pnuemonia and Meningitis are all life-threatening diseases, but you can protect your child from them by immunising him/her. The first immunisation dose is administered to your child right after birth, but there are four other doses that must be administered in the first year. Make sure your child gets all the due immunisation from the health facility which are available free of cost under National Immunization Programme. Your child will be completely protected only when he gets all four doses. Missing even one dose results in a risk of illness. Some vaccination can have minor side effects like redness, mild fever, which are normal and go away after a while. If you're

		concerned about the side effects contact your ASHA/ANM or visit the nearest health center.
**34**	IUCD	Are you worried about getting pregnant again or thinking about how to limit your family's size? Are you wondering about which method will prevent pregnancy right now but will still allow you to have a child later? The IUCD is a simple and effective solution to your worries. There are two kinds of IUCDs - one that lasts for 5 years and one that lasts for 10 years. It can be inserted at a health facility by a trained doctor or nurse 6 weeks post delivery. And remember you can have it removed whenever you want to have a baby. The IUCD is inserted for free. Talk to your ASHA/ANM about choosing an IUCD today.
**35**	Exclusive breastfeeding, WASH	Children often fall ill because of a lack of cleanliness and hygiene. And this places them at the risk of repeatedly getting diarrhoea and pneumonia. This prevents the child from attaining height and weight appropriate for its age and impacts the child's mental development. Protecting your child is easy with these two steps - first, exclusive breastfeeding for six months and second, everyone should wash their hands with soap and water after defecation. Make sure you also wash your hands before cooking or handling food and before feeding the baby. Also wash your hands after cooking and after feeding your baby. This habit will protect your child from infection. If your child falls ill and is irritable and doesn't want to breastfeed, make sure you still keep feeding it milk at regular intervals. This will help your child get better quickly.
**36**	Immunization	When your child turns three and a half months old, make sure you take it for its next immunisation dose. Some vaccinations require multiple doses before they are effective. Make sure your child gets every dose of every vaccination. If for some reason you've missed a previous dose, make sure your child gets those too. Ask your ASHA/ANM about this. You do not have to go far to get your child vaccinated. Just take your child to the nearest health center. And remember to carry your MCP card - mother and child protection card with you. So remember to get your child vaccinated at three and a half months.
**37**	Child development	Your baby must be growing quickly and you may have noticed that your baby has started doing new things. Interact with the baby - regularly talking and playing with the baby help to develop both body and mind. Any way baby will grow according to age, but it is important to know whether the development is happening properly or not. So for this And remember to make sure you ask the ASHA/ANM/AWW about the Rashtriya Bal Swasthya Karyakram screening at anganwadis and the free services available under this scheme. It entitles you to free check-ups for six years to assess your baby's physical and mental growth and development.
**38**	Pneumonia	Young children can sometimes fall ill, but always remember that some diseases are life threatening so pay attention to the child's symptoms. One such life threatening disease is Pneumonia. If your child has fever with chest in drawing (tez pasli chalna), difficulty breathing, not drinking milk, this could be pneumonia. Consult the ASHA/ANM/ nearest health facility immediately and get their advice as soon as you spot the symptoms of pneumonia. Don't waste time, because right medication at the right time can save your baby's life from pneumonia.
**39**	Diarrhoea WASH	Diarrhoea causes dehydration that can be life threatening for babies. Getting into the habit of cleanliness and regular hand-washing will help prevent diarrhoea and also helps in baby's development. After defecation and before feeding your baby, make sure you wash your hands. Feed the baby out of a clean utensil. Also keep the following two things in mind - first, make sure you exclusively breastfeed for six months and start appropriate complementary feeding after 6 months; second, immunize your baby completely.

**40**	Child development	Remember all babies grow differently and reach their developmental milestones at their own pace. If you feel the baby is not developing as it should, get your child checked up under the RBSK. The RBSK gives you free check-ups for six years to test your child's mental and physical development. Your baby is now 4 months old, so it must have started doing new things like being able to bring both hands together, lifting its head, keeping its hands open and relaxed, responding to sounds, maintaining eye contact and smiling. Interact with the baby - this will help to develop the baby's body and mind. If your baby seems underdeveloped, ask your ANM/Anganwadi worker about the RBSK immediately.
**41**	Diarrhoea	Young children can sometimes fall ill, but always remember that some diseases are life threatening. One such life threatening disease is diarrhoea, which causes dehydration. It also affects the physical and mental development of the baby. If the baby has more than usual number of stools, or more watery stools than usual, it could be diarrhoea. Immediately start feeding your baby ORS solution and meet your ASHA/ANM. If you delay treatment, your child will be severely dehydrated and its life will be in danger.
**42**	Immunization	All immunisation doses are important and should not be missed. Getting your child vaccinated will prevent him/her from life-threatening diseases. Missing even one dose cause can open up chances of getting diseases. Always remember that a child will be completely protected from the vaccine preventable diseases if all immunisation doses are administered. Mother and Child Protection (MCP) card will help you in tracking all the due vaccinations. If you have not got your MCP card yet, please contact your ASHA or ANM for the same. Make sure your child gets all the due immunisation from the health facility which are available free of cost under Universal Immunization Programme.
**43**	Growth monitoring	You can't always tell by looking at a child whether it is getting enough nutrition and growing well and thus regular weighing is important. At Anganwadi centre or on the monthly village VHSND or immunisation day ask your AWW or ANM to weigh your child and record it the MCP (mother and child protection) card. In case your baby's weight is less as per age (underweight) and needs extra nutrition , you can get extra food at the AWC. You will also get advice about what and how to feed your baby so that she/he grows well.
**44**	Child anaemia IFA	Deficiency of iron in your children leads to anemia and prevents them from being active, learning and playing. Iron is very important for the physical and mental development of your child. Your child requires IRON FOLIC ACID syrup twice a week from 6 months up to 5 years of age. Ask your ASHA/ANM for iron syrup bottle which is available free of cost. Iron helps in development of brain and body. So don't hesitate - ask your ASHA/ANM about this.
**45**	Diarrhoea zinc IYCF	Diarrhoea causes dehydration that can be life threatening for babies. Getting into the habit of cleanliness and regular hand-washing will help prevent diarrhoea and also helps in baby's development. After defecation and before feeding your baby, make sure you wash your hands. Feed the baby out of a clean utensil. Also keep the following two things in mind - first, make sure you exclusively breastfeed for six months and start appropriate complementary feeding after 6 months; second, immunize your baby completely.
**46**	Diarrhoea zinc	Diarrhoea causes dehydration and can be life-threatening. If your baby has diarrhea immediately start giving it ORS with potable water. For babies over six months also give zinc to the baby for 14 days. Contact your ASHA or ANM immediately who will be able to advise you on what you should do. ORS with potable water helps in preventing dehydration which is life threatening. Use of zinc for 14 days, Increased/extra feeding, including breast feeding help the baby to recover faster, builds immunity and make up for loss of growth.

**47**	IYCF thick	As soon as the baby completes 6 months is start feeding semi-solid mashed food to the baby. Feed your child whatever you make at home - just mash it and add ghee or oil. Start to feed semi-solid mashed food with oil/ghee. The baby will be able to eat even if it doesn't have teeth. Feed the baby three times a day and continue breastfeeding as well. This ensures that the baby gets adequate nutrition for the growth of its body and mind and gives the baby power to fight critical illness. And always remember to wash your hands before you prepare food and feed the baby.
**48**	Child development	Your baby has completed 6 months, and may have now has started to hold its head straight while sitting or when held. S/he can most likely follow an object and may be trying to reach for it and grasp it. Make sure to interact with the baby as much as possible - this will help to develop the baby's body and mind. Remember all babies grow differently and reach their developmental milestones at their own pace. If you feel the baby is not developing as it should, get it checked under the RBSK immediately. Under the RBSK, children under 6 years of age can get their physical and mental development checked for free. If you think your child's development is lacking, ask your ASHA/ANM/AWW about RBSK immediately.
**49**	IYCF thick, WASH	Your child is now older than six months and so must be eating food other than breast milk. Don't feed your child liquid food like daal ka paani. Make sure whatever you feed to your baby is thick in consistency so that it doesn't flow through your fingers. Your baby will be stronger and more energetic if you feed it thick food. If the food is too watery then your baby may not get adequate nutrition. Remember to wash hands while preparing the food and before feeding the baby. Remember to give more food to the baby during illness- this helps the baby to recover faster.
**50**	IYCF complementary feeding	Your child is now older than six months and so must be eating food other than breast milk. Feed your child 3-4 times a day and also give it breastmilk. Feed your child whatever you make at home - just mash it and add ghee or oil. Daal-roti and sabzi are very nutritious. Also feed your child kheer, porridge, sevai, boiled potatoes and fruit. Wash your own hands with soap and water every time before and after cooking, storing or feeding.
**51**	IYCF & WASH	Your child is now older than seven months and so must be eating food other than breast milk. S/he probably puts whatever s/he can find in his/her mouth. This is why the risk of illness or infection is greater right now. But two small habits can help keep your baby healthy - first, make sure your environment is clean, and second, give your baby safe drinking water. Also remember to cover the water and food - don't leave it open because flies can sit on it and spread disease. Remember unclean water is the root cause of deadly diseases like diarrhoea so make sure you only feed clean drinking water.
**52**	IYCF portions, thick	You need to actively feed mashed and thick food, and monitor what the baby is eating. Make sure to feed the baby in a separate clean katori. If you actively feed the baby in a separate katori then you can tell how much the child is eating. It will ensure that the baby is eating enough and won't get hungry soon. Your baby will be irritable and eat less if it is ill, but you must make sure to feed it small amounts regularly so that it recovers quickly.
**53**	Pregnancy test	You have not used any family planning methods and Have you missed your periods? Anxious about your pregnancy? Don't Worry! The easy to use 'Nishchay' pregnancy test kit is now available with ASHAs and at sub health centres free of cost. The kit will tell you immediately if you are pregnant. If your pregnancy is confirmed, make sure you get a health check-up.
**54**	Sterilization	Being well looked-after, well fed and educated is every child's right. But this becomes difficult if your family becomes too big. Times have changed, and it's best to have a happy family, small family. Talk to each other and decide upon a permanent family planning method either male or female sterilization. Both

		services are safe and reliable. A small family will result in savings and a brighter future. Ask your ASHA/ANM about this today.
**55**	Male sterilization	Male sterilisation is an easy way to control the size of your family. It is a permanent, simple, quick, one-time in procedure and can take rest in whole life. No incision or stitches are given in the procedure and it does not require hospital stay. Male sterilisation doesn't result in weakness and it doesn't affect married life. It is an easier procedure than that for female sterilization. It is free in government hospitals, and the government compensates you for your loss of wages on that day. Contact your ASHA or ANM for more information today.
**56**	Immunization	Your child is growing well, so don't forget to celebrate by getting your child its next vaccination. When your child completes nine months, it is time for another immunisation dose. As I said earlier, missing even one does means putting your child in danger of life-threatening diseases. Make sure you go for the vaccination on the teekakaran divas in your nearest health facility. Make sure you carry your MCP (mother and child protection) card along with you. The ANM will also give your baby Vitamin A syrup which will help make your baby strong and will maintain good eyesight for your baby. If for some reason you've missed a previous dose, make sure that it's also administered. Ask your ASHA/ANM about this.
**57**	Malaria	If your child has fever, don't take it lightly because it could be malaria. Malaria is a life threatening condition for children if not treated on time. If the child has fever consult ASHA/ANM or nearest health centre for diagnosis of malaria and treatment. If your baby has malaria, have it treated immediately. Follow the doctor's advice and prescription for early recovery of your baby.
**58**	Female sterilization	If you feel that you don't want to have any more children, you can go for female sterilization. This can be done within 7 days of delivery, or you can ask your ASHA/ANM and decide when to get it done. Female sterilization is a one-time, permanent method of contraception for women. Female sterilisation is performed for free at government hospitals and the government will compensate you for loss in wages on that day. Ask your ASHA/ANM about this today.
**59**	Diarrhoea	Diarrhoea causes dehydration and can be life-threatening. If your baby has diarrhea immediately start giving it ORS with potable water. For babies over six months also give zinc to the baby for 14 days. Contact your ASHA or ANM immediately who will be able to advise you on what you should do. ORS with potable water helps in preventing dehydration which is life threatening. Use of zinc for 14 days, Increased/extra feeding, including breast feeding help the baby to recover faster, builds immunity and make up for loss of growth.
**60**	Spacing	Remember - keep a gap of at least three years between the first and second child. Remember the mantra 1 3 2 means three years gap between two children. This is beneficial because you will be able to care for, educate and financially support your child, which will give it a better future. Keeping a gap between pregnancies is good for the health of both mother and baby. The mother gets appropriate time to restore her health and energy and the next baby will be born healthy too. Talk to each other and contact your ASHA/ANM soon to know methods of spacing.
**61**	Pneumonia	Young children can sometimes fall ill, but always remember that some diseases are life threatening so pay attention to the child's symptoms. One such life threatening disease is Pneumonia. If your child has fever with chest in drawing (tez pasli chalna), difficulty breathing, not drinking milk, this could be pneumonia. Consult the ASHA/ANM/ nearest health facility immediately and get their advice as soon as you spot the symptoms of pnuemonia. Don't waste time, because right medication at the right time can save your baby's life from pneumonia.

**62**	Malnutrition signs	I hope you're feeding your baby well, because a lack of food results in malnutrition. Malnutrition weakens the immune system (bimariyon se ladne ki takat kam karta hai), which results in children falling sick frequently. It could also be life-threatening, so don't treat it lightly. Your child could be malnourished if you notice any of the following symptoms: Loss of appetite in the baby, difficulty putting on weight or loss of weight, frequent diarrhoea, extremely thin arms and legs and/or swelling in both feet and change in hair colour (brown). Immediately contact your ASHA/ANM/AWW for advice on what to do in case you notice any of these symptoms.
**63**	Child anaemia	Deficiency of iron in your children impacts development, because it impacts the brain and the body. In case the palms of your child's hands are yellow, the child is sick/weak and less active, or feels tired often, ask the ANM/nearest health centre to test your child for anaemia. If your child is anaemic, feed him/her iron syrup. Iron syrup is available free of cost with ASHA/ANM or nearest health centre. Make sure you ask when and how much to feed your child.
**64**	IYCF iron rich foods	Iron deficiency impacts your child's body and mind, and causes your child to be weak and less active. Improve iron content of the child's diet by giving iron rich foods to them every day. Iron rich foods include green leafy vegetables like spinach (palak), amaranth (chaulai sag/dantu), soyabean, green vegetables, bengal gram (kala chana) turnip leaves (shalgam ka sag), mint leaves (pudhina), small amounts of tamarind (imli), lemon, fruit like guava and water melon and dates figs if possible. If you are non-vegetarian, feed your child meat and fish as well. If you still feel that your child is anaemic, speak to your ASHA/ANM immediately.
**65**	Oral contraceptive pills	Worried about unwanted pregnancy? Govt of India has launched a new tablet, called *Chaaya.* You can take this pill even if you are breastfeeding or thereafter. There is no negative impact on your body with this pill. You can start taking these pills if you are not pregnant. You can get these new pills *Chaaya* or other OCPs from health centers or from your ASHA. It might affect your menstrual cycle in the beginning but don't worry it means that the pill is having the desired effect. You just need to ensure that they are taking the right dose of *Chaaya* at the right time and in the right way. If you face any problem after taking the pill talk to your ASHA.
**66**	Condoms	If you think how to keep a small family. If you're looking for a quick and handy family planning method then consider the condom. The condom is an easy to use and safe contraceptive method for men which protects you from unplanned pregnancy as well as sexually transmitted infections and HIV. But, it is very important for you to remember to use a condom every time you have sex in order for it to be effective. Make it a habit and you can stop worrying about unwanted pregnancies. Condoms are available at any government hospital and with ASHAs. Contact your ASHA or ANM for more information.
**67**	WASH	You cook food, eat and feed the child with your hands and the hands often have germs that are invisible to the naked eye, which cause sickness and unnecessary spending. Start washing your hands with soap to avoid this. Make sure you wash your hands after going to the toilet, before cooking food and before eating or feeding your baby. Keep soap and water in your bathroom and kitchen so you remember to wash your hands. Illness will be away when hands are clean and beautiful.
**68**	Emergency contraceptive pills	If you are in a situation where you had forget to take pill or use condom (unprotected sex) and are now worried that you may be pregnant, then you have an option - the emergency contraceptive pill. The Emergency Contraceptive Pill helps prevent pregnancy when taken within 72 hours of unprotected sex, but it is ineffective after 72 hours. But remember, the EC pill is not a regular contraceptive method and should be used only in case of an emergency. Contact your ASHA or ANM for more information.

**69**	IYCF, frequency potion, diverse, WASH	Now your child is one year old, breastfeed as often as the child wants. Give complementary food at least 5 times a day. You can feed your child whatever you make at home, such as dal-roti, green vegetables, kheer, sevai, porridge, boiled potatoes and fruit. Give at least 1 and a half katori serving at a time. If you eat eggs, chicken, meat or fish, also feed this to your baby. Wash your own hands with soap and water every time before and after feeding.
**70**	Child development	Remember all babies grow differently and reach their developmental milestones at their own pace. If you feel the baby is not developing as it should, get your baby checked under the RBSK. The RBSK provides free check-ups for the physical and mental development of your child until it turns six years old. Your baby may have started to walk with external support, hold objects in its hands and throw or play with them, or put them in his/her mouth. Your baby may have started shaking its head 'yes' or 'no'. Spend time with your child. If you feel like your child has not developed enough, ask your ANM/AWW about RBSK immediately.
**71**	Immunization	Immunisation is your biggest responsibility, because it protects your baby from life-threatening diseases which can be prevented by vaccines. When the baby turns 1.5 years old make sure to go for the next dose of immunization, which are measles and polio booster Make sure you go for the vaccination on the teekakaran divas in your nearest health facility. The ANM will also give your baby Vitamin A syrup which will help make your baby strong and will maintain good eyesight for your baby. So don't forget to have your child immunised when it turns one and a half years old. If for some reason you've missed a previous dose, make sure that it is also administered. Ask your ASHA/ANM about this.
**72**	Goodbye	This is the last message from the Government of India's Kilkari mobile service. I will not be there from next week, but remember that the ASHA, AWW and ANM are your friends. Seek advice from them whenever you need any information. Remember it is much better to seek advice when you are unsure and take action right away if needed. Take your family's health in your hands. This will ensure the health and happiness of the child, the mother and the family. Good luck and goodbye.
