# Supplementary material for: Another voice in the crowd: the challenge of changing family planning and child feeding practices through mHealth messaging in rural central India
Source: BMJ Glob Health. 2021 Jul 26;6(Suppl 5):e005868. doi: 10.1136/bmjgh-2021-005868 (PMC8327813; doi:10.1136/bmjgh-2021-005868)
Supplement: Supplementary data [file bmjgh-2021-005868supp002.pdf]

BMJ Publishing Group Limited (BMJ) disclaims all liability and responsibility arising from any reliance placed on this supplemental material which has been supplied by the author(s)

Supplementary file 2. Listening profiles for each subscriber's mobile number (n=29), by Kilkari weekly call

WEEKLY MESSAGE / HOUSEHOLD KILKARI SUBSCRIBER ID	w1 Welcome	w17 Immediate breastfeeding	w18 Immunization	w19 Postpartum checkup	w20 Postpartum IUCD, female sterilization	w21 Thermal Care	w22 Cord care	w23 Immediate breastfeeding	w24 IUCD	w25 Postpartum checkup WASH	w26 Exclusive breastfeeding	w27 Child danger signs	w28 Immunization	w29 MPA injection	w30 Free healthcare for child	w31 Exclusive breastfeeding	w32 Immunization	w33 Immunization	w34 IUCD	w35 Exclusive breastfeeding, WASH	w36 Immunization	w37 Child development	w38 Pneumonia	w39 Diarrhoea WASH	w40 Child development	w41 Diarrhoea	w42 Immunization	w43 Growth monitoring	w44 Child anaemia IFA
WOM_01	0-24%	25-49	75%+	75%+	75%+	75%+	75%+		0-24%	25-49	75%+	75%+	25-49	75%+	0-24%	0-24%	75%+		75%+	75%+	75%+	0-24%	75%+	75%+	75%+		75%+	0-24%	0-24%
WOM_02	75%+									25-49	25-49	75%+	50-74	50-74	0-24%	0-24%	0-24%	0-24%	0-24%	50-74	0-24%	75%+	75%+	75%+	50-74	75%+	75%+	75%+	75%+
WOM_03	75%+		75%+	75%+	75%+	75%+	75%+	75%+	75%+	25-49	0-24%	75%+	75%+	0-24%	25-49	75%+	75%+	75%+	75%+	75%+	0-24%	75%+	75%+	0-24%	75%+	75%+	0-24%	0-24%	0-24%
WOM_04	75%+	75%+	0-24%	0-24%	0-24%	0-24%	75%+	0-24%	0-24%	0-24%	25-49	0-24%	0-24%	75%+	0-24%	75%+	25-49	0-24%	0-24%	75%+	0-24%	75%+	0-24%	0-24%	0-24%	75%+	75%+	75%+	75%+
WOM_05	0-24%	75%+	75%+	75%+	75%+	75%+	75%+	25-49	75%+	75%+	25-49	75%+	75%+	75%+	75%+	75%+		0-24%	75%+	75%+	75%+	75%+	75%+	75%+	75%+	75%+	75%+	75%+	75%+
WOM_06	75%+		75%+	75%+	75%+	75%+	75%+	75%+	75%+	75%+	75%+	75%+	75%+	50-74	75%+	0-24%	75%+				75%+	75%+	75%+	75%+	75%+	75%+	75%+	75%+	75%+
WOM_07	75%+			75%+	0-24%	75%+	0-24%	25-49	75%+	75%+	75%+	75%+	25-49	0-24%	75%+	25-49	75%+	75%+	0-24%	75%+	75%+	75%+	75%+	75%+	75%+	75%+	75%+	75%+	75%+
WOM_08	75%+	75%+		75%+	75%+	25-49	0-24%	75%+		50-74	75%+	75%+	75%+	50-74	0-24%	75%+	75%+	75%+	75%+	75%+		25-49	75%+	75%+	75%+	75%+	50-74	75%+	75%+
WOM_09	75%+					75%+	75%+	25-49	75%+	75%+	75%+	75%+	75%+	75%+	75%+	75%+	75%+	75%+	75%+	75%+	75%+	75%+	75%+	75%+	75%+	75%+	75%+	75%+	75%+
WOM_10	75%+						75%+	0-24%	0-24%	0-24%	75%+	75%+	75%+	75%+	75%+	75%+		75%+	0-24%	75%+	75%+	75%+	75%+	75%+	75%+	75%+	75%+	75%+	75%+
WOM_11	75%+								75%+	75%+	75%+	75%+	25-49	0-24%	0-24%	75%+	50-74	75%+	0-24%	0-24%	0-24%	0-24%	75%+	75%+	75%+	75%+	0-24%	0-24%	50-74
WOM_12	75%+				75%+	75%+	75%+	0-24%	75%+	75%+	75%+	75%+	0-24%	75%+	75%+	75%+				0-24%	75%+	75%+	75%+	75%+	50-74	75%+	75%+	75%+	75%+
WOM_13		75%+	75%+	75%+	75%+	75%+	75%+	75%+	75%+	75%+	75%+	75%+	75%+	75%+	75%+	75%+								75%+	75%+	75%+	50-74	75%+	75%+
WOM_14	75%+				0-24%	75%+	0-24%	75%+	75%+	75%+	0-24%	75%+	75%+	0-24%	75%+	75%+	75%+	0-24%	0-24%	75%+	0-24%	0-24%	75%+	75%+	0-24%	75%+	25-49	75%+	75%+
WOM_15	75%+				75%+	75%+	75%+	25-49	50-74	75%+	75%+	75%+	75%+	0-24%	50-74	75%+	75%+	0-24%	0-24%	0-24%	25-49	75%+	75%+	75%+	75%+	75%+	75%+	75%+	75%+
WOM_16	0-24%						75%+	75%+	75%+	75%+	25-49	75%+	75%+	75%+	75%+	25-49					0-24%	25-49	0-24%	0-24%	25-49	75%+	0-24%	0-24%	0-24%
WOM_17	75%+		75%+	75%+	75%+	75%+	75%+	0-24%	25-49	0-24%	0-24%	75%+	75%+	75%+	75%+	75%+	75%+	75%+	75%+	0-24%	75%+			25-49	0-24%	0-24%	0-24%	25-49	25-49
WOM_18	75%+							0-24%	25-49	0-24%	25-49	0-24%		0-24%	75%+	0-24%	75%+	75%+		25-49	0-24%		75%+	75%+	25-49			75%+	75%+
WOM_19	75%+	75%+	25-49	75%+	75%+	75%+	75%+	75%+	75%+	75%+	75%+	75%+	75%+	75%+	75%+	75%+	75%+	75%+	50-74	75%+	75%+	0-24%	75%+	75%+	0-24%	75%+	75%+	25-49	25-49
WOM_20	0-24%					75%+	75%+	75%+	0-24%	75%+	75%+	25-49	75%+	75%+	75%+	75%+	75%+	75%+	0-24%	25-49					0-24%	0-24%	75%+		
WOM_21	75%+			75%+	75%+	75%+	75%+	75%+	75%+	75%+	75%+	75%+	75%+	75%+	75%+	75%+		75%+	75%+	75%+	75%+	75%+	75%+	75%+	75%+	75%+	75%+	75%+	75%+
WOM_22	75%+				75%+	75%+	0-24%	50-74	75%+	75%+	75%+	75%+	75%+	75%+	75%+	75%+	75%+	75%+	75%+	75%+	75%+	75%+	75%+	75%+	75%+	75%+	75%+	75%+	75%+
WOM_23	75%+	75%+	75%+	75%+	25-49	75%+	0-24%	75%+	75%+	0-24%	75%+	75%+	75%+	0-24%					0-24%	0-24%	0-24%	0-24%	0-24%	25-49	75%+				75%+
WOM_24	75%+			75%+	75%+	75%+	0-24%	75%+	75%+	75%+	50-74	75%+	75%+	75%+	75%+	75%+	75%+	75%+	75%+	75%+	0-24%	75%+	75%+	75%+	75%+	75%+	75%+	75%+	0-24%
WOM_25	0-24%			25-49	75%+	75%+	75%+	75%+	0-24%	50-74	0-24%	75%+	75%+	75%+	0-24%	75%+	50-74		0-24%	25-49	75%+	0-24%	0-24%	0-24%	75%+	0-24%	75%+	50-74	75%+
WOM_26	75%+	0-24%	75%+	75%+	75%+	75%+	75%+	75%+	75%+	75%+	75%+	75%+	75%+	75%+	75%+	75%+	75%+	75%+		75%+	75%+		75%+	75%+	75%+	75%+	75%+	75%+	75%+
WOM_27		0-24%	25-49	75%+	75%+	50-74	25-49	25-49	0-24%	75%+	50-74	75%+	50-74	75%+	75%+		0-24%	75%+	25-49	75%+	75%+	75%+					0-24%	0-24%	75%+
WOM_28	0-24%							75%+	75%+	75%+	50-74	75%+	75%+	0-24%	75%+	0-24%						75%+	75%+	75%+	0-24%	75%+	75%+	75%+	75%+
WOM_29	50-74	0-24%	75%+	75%+	75%+	75%+	25-49	0-24%	0-24%	75%+	75%+	75%+	25-49	25-49	75%+	25-49	75%+	25-49	75%+	75%+	75%+	75%+	50-74	75%+		25-49	25-49	50-74	0-24%

WEEKLY MESSAGE / HOUSEHOLD KILKARI SUBSCRIBER ID	w45 Diarrhoea zinc IYCF	w46 Diarrhoea zinc	w47 IYCF thick	w48 Child development	w49 IYCF thick, WASH	w50 IYCF complementary feeding	w51 IYCF & WASH	w52 IYCF portions, thick	w53 Pregnancy test	w54 Sterilization	w55 Male sterilization	w56 Immunization	w57 Malaria	w58 Female sterilization	w59 Diarrhoea	w60 Spacing	w61 Pneumonia	w62 Malnutrition signs	w63 Child anaemia	w64 IYCF iron rich foods	w65 Oral contraceptive pills	w66 Condoms	w67 WASH	w68 Emergency contraceptive pills	w69 IYCF, frequency potion, diverse, WASH	w70 Child development	w71 Immunization	w72 Goodbye
WOM_01	75%+	0-24%	0-24%	75%+	0-24%	75%+	75%+	75%+	75%+	50-74	75%+	75%+	0-24%	0-24%	0-24%	75%+	0-24%	0-24%	0-24%	0-24%	75%+	0-24%		25-49				0-24%
WOM_02			75%+	75%+	75%+	75%+	75%+	75%+	75%+	50-74	75%+			75%+	0-24%	75%+	75%+	75%+	75%+	75%+	75%+	25-49	75%+	75%+	75%+	75%+	75%+	75%+
WOM_03	0-24%	0-24%	75%+	75%+	75%+	75%+	75%+	0-24%	75%+	75%+	75%+	75%+	75%+	75%+	75%+	75%+	75%+	75%+	75%+	75%+	75%+	75%+	75%+	75%+	75%+	75%+	75%+	75%+
WOM_04	75%+	75%+	75%+	75%+	0-24%	75%+	75%+	0-24%	75%+	75%+	0-24%	50-74	0-24%	75%+	25-49	25-49	0-24%	75%+	0-24%		75%+	0-24%	0-24%	0-24%	0-24%	75%+	50-74	75%+
WOM_05	75%+	75%+	75%+	75%+	75%+	75%+	75%+	75%+	75%+	75%+	75%+	75%+	75%+	25-49	75%+	75%+	75%+	75%+	75%+	75%+	75%+	25-49	50-74	75%+	75%+	75%+	75%+	75%+
WOM_06	75%+			75%+	25-49	75%+	0-24%	75%+	75%+	75%+	0-24%	0-24%			75%+	75%+	75%+	75%+	75%+	0-24%	75%+	75%+	25-49	75%+		50-74	0-24%	0-24%
WOM_07	75%+	75%+	75%+	75%+	75%+	75%+	75%+	75%+	75%+	75%+	0-24%	75%+	75%+										75%+	75%+			75%+	
WOM_08	75%+	75%+	75%+	0-24%	75%+	0-24%	75%+	75%+	0-24%	75%+	75%+																	
WOM_09	75%+	75%+	25-49	75%+	75%+	75%+	75%+	75%+	75%+	75%+	75%+	75%+	75%+	75%+	75%+	75%+	75%+	75%+	0-24%	75%+	75%+	75%+	75%+	75%+	75%+	75%+	75%+	75%+
WOM_10	75%+	75%+	75%+	75%+	75%+	75%+	75%+	75%+	75%+	75%+	75%+	75%+					75%+											
WOM_11		0-24%	75%+			75%+	75%+	0-24%	25-49	75%+	75%+	0-24%	75%+	75%+			0-24%	25-49		75%+	75%+		0-24%	75%+	25-49	75%+	75%+	75%+
WOM_12	0-24%	25-49	75%+	75%+	75%+	75%+	75%+	0-24%		75%+	75%+	75%+	75%+	75%+	75%+	75%+	75%+	75%+	75%+	25-49	75%+	75%+	25-49	75%+	75%+	75%+		
WOM_13	50-74	75%+	75%+	75%+	75%+	0-24%	75%+	75%+	75%+	25-49	75%+	75%+	75%+	0-24%	75%+	75%+	75%+											
WOM_14	0-24%	75%+	75%+	75%+	75%+	75%+	75%+	75%+	0-24%	75%+	25-49	75%+	0-24%	0-24%	0-24%	75%+	75%+	75%+	75%+	0-24%	75%+	0-24%	75%+	75%+				0-24%
WOM_15	75%+	75%+	50-74	75%+	0-24%	50-74	75%+	75%+	25-49	75%+	25-49	50-74	75%+	75%+	50-74	75%+	75%+	75%+	75%+	75%+	50-74	0-24%	75%+	0-24%	25-49	75%+	75%+	75%+
WOM_16	25-49	25-49	0-24%	0-24%	75%+	0-24%	75%+	75%+	75%+	75%+	75%+	75%+	75%+	25-49				0-24%	75%+	0-24%	0-24%	75%+	75%+		0-24%	75%+	75%+	
WOM_17	25-49	0-24%	0-24%	0-24%			75%+	75%+	75%+		75%+		50-74		75%+	75%+		25-49	75%+	25-49	0-24%		75%+			75%+		
WOM_18	75%+	75%+				75%+	75%+	0-24%	0-24%	75%+	25-49		75%+			75%+	75%+	0-24%										
WOM_19	50-74	75%+		0-24%	75%+	75%+	75%+	75%+	75%+	75%+	75%+	75%+	50-74	0-24%	75%+	75%+	75%+		75%+	0-24%	0-24%	75%+	0-24%	75%+		50-74	75%+	
WOM_20	75%+	75%+	75%+	75%+	75%+	75%+	0-24%	75%+	75%+	75%+		75%+	75%+	0-24%		25-49	75%+	75%+	75%+	75%+	75%+	75%+	75%+	75%+	0-24%	75%+	75%+	75%+
WOM_21	75%+	75%+	75%+			75%+	75%+	75%+	75%+	75%+	75%+	75%+	75%+	0-24%	75%+						75%+	75%+	75%+	75%+	75%+	75%+	75%+	75%+
WOM_22	75%+	75%+	75%+	75%+	75%+	75%+	75%+	75%+	75%+	75%+	75%+	75%+	75%+	75%+	25-49	75%+	0-24%		75%+	75%+	75%+	75%+	75%+	75%+	75%+	75%+	50-74	75%+
WOM_23	0-24%	0-24%	75%+	75%+	0-24%	0-24%	0-24%		75%+	0-24%	75%+	75%+	0-24%	0-24%	0-24%	25-49	75%+	0-24%	75%+	0-24%	0-24%	25-49	0-24%	0-24%	75%+	75%+	75%+	75%+
WOM_24	0-24%	75%+	75%+	75%+	75%+	75%+	75%+	75%+	75%+	75%+	75%+	75%+	75%+	75%+	75%+	50-74	75%+	75%+	75%+	75%+	25-49	75%+	75%+	75%+	75%+	75%+	75%+	75%+
WOM_25	0-24%	0-24%	75%+	75%+	75%+	75%+		75%+	0-24%	75%+	75%+	75%+	25-49	75%+	0-24%	0-24%	0-24%		0-24%	0-24%	75%+	75%+	75%+				75%+	75%+
WOM_26			75%+	50-74	75%+	75%+	75%+	75%+	75%+	75%+	75%+	75%+	25-49	75%+	50-74	75%+	25-49	75%+	75%+	75%+	75%+	25-49	75%+	75%+	75%+	75%+	75%+	75%+
WOM_27	0-24%	0-24%	0-24%	50-74					0-24%		0-24%	75%+	0-24%	0-24%	0-24%	75%+	0-24%											
WOM_28	75%+	0-24%	0-24%	75%+	50-74	0-24%	0-24%	25-49	75%+	0-24%	75%+	75%+	75%+	0-24%	75%+	25-49	75%+	25-49	75%+	0-24%	0-24%	75%+	0-24%	75%+	0-24%	75%+	0-24%	75%+
WOM_29	75%+	75%+	25-49		75%+	75%+	75%+	75%+	75%+	75%+	25-49	75%+	0-24%	75%+	75%+	75%+	75%+	75%+	0-24%	75%+	75%+	75%+	25-49	75%+		75%+		

The columns indicate the weekly messages, starting with week 1, which is the introduction message that goes out to all subscribers in their first week. Week 17 indicates the Kilkari call sent out during the first week of the eighth month of pregnancy; many subscribers miss the first 16 weeks of Kilkari because of late enrollment, so we focused on weeks 17 to 72 to mimic current patterns of likely exposure. The child's birth takes place around week 25. The child turns one and the parents "age out" of Kilkari at week 72.

	The red cell indicates the last message that the subscribed mobile phone picked up before our qualitative research team met with them for the interview.
	The grey cells indicate all messages that the subscriber had not been in Kilkari long enough to receive yet, at the time of the interview.
75%+	The dark green cells indicate calls that were picked up and listened to for at least 75% of their total duration (e.g., for a 90 second call, the subscriber listened to at least 67.5 seconds)*
50-74%	The mid-green cells indicate calls that were picked up and listened to for between 50 and 74% of their total duration*
25-49%	The light green cells indicate calls that were picked up and listened to for between 25 and 49% of their total duration
0-24%	The light yellow cells indicate calls that were "cut", i.e. picked up and listened to for less than 25% of their total duration
	The blank cells represent calls that were never picked up, either because the phone did not ring (out of network, switched off, etc.) or because it rang but was not picked up.
* Any call listened to for 50% or more of its duration is considered to have been "listened to" overall, because the key health content would have been delivered	
